# Sodium chloride promotes macrophage pyroptosis and aggravates rheumatoid arthritis by activating SGK1 through GABA receptors Slc6a12

**DOI:** 10.7150/ijbs.93242

**Published:** 2024-05-11

**Authors:** Xianzheng Zhang, Ziwei Zhang, Yuchen Zhao, Lin Jin, Yu Tai, Yujing Tang, Shuo Geng, Han Zhang, Yufang Zhai, Yining Yang, Pin Pan, Peng He, Shuqi Fang, Chenlong Sun, Yu Chen, Mengqi Zhou, Lianghu Liu, Han Wang, Li Xu, Tianjing Zhang, Jinghan Hua, Hua Wang, Lingling Zhang

**Affiliations:** 1 Department of Oncology, The First Affiliated Hospital of Anhui Medical University, Hefei, China.; 2 Institute of Clinical Pharmacology, Anhui Medical University, Hefei, China; Key Laboratory of Anti-inflammatory and Immune Medicine, Ministry of Education, Hefei, China; Anti-inflammatory Immune Drugs Collaborative Innovation Center, Anhui Province, Hefei, China.; 3 Department of orthopedics, The Second People's Hospital of Hefei, Hefei Hospital Affiliated to Anhui Medical University, Hefei, Anhui 230011, China.; 4 Department of Orthopedics, First Affiliated Hospital of Anhui Medical University, Hefei, China.

**Keywords:** High-salt diet, rheumatoid arthritis, macrophages, pyroptosis, SGK1, Slc6a12

## Abstract

Rheumatoid arthritis (RA) is a chronic systemic autoimmune disease characterized by synovial inflammation and the production of autoantibodies. Previous studies have indicated an association between high-salt diets (HSD) and an increased risk of RA, yet the underlying mechanisms remain unclear. Macrophage pyroptosis, a pro-inflammatory form of cell death, plays a pivotal role in RA. In this study, we demonstrate that HSD exacerbates the severity of arthritis in collagen-induced arthritis (CIA) mice, correlating with macrophage infiltration and inflammatory lesions. Given the significant alterations observed in macrophages from CIA mice subjected to HSD, we specifically investigate the impact of HSD on macrophage responses in the inflammatory milieu of RA. In our *in vitro* experiments, pretreatment with NaCl enhances LPS-induced pyroptosis in RAW.264.7 and THP-1 cells through the p38 MAPK/NF-κB signaling pathway. Subsequent experiments reveal that Slc6a12 inhibitors and SGK1 silencing inhibit sodium-induced activation of macrophage pyroptosis and the p38 MAPK/NF-κB signaling pathway, whereas overexpression of the SGK1 gene counteracts the effect of sodium on macrophages.

In conclusion, our findings verified that high salt intake promotes the progression of RA and provided a detailed elucidation of the activation of macrophage pyroptosis induced by sodium transportation through the Slc6a12 channel.

## Introduction

Rheumatoid arthritis (RA) is a chronic systemic disorder defined by synovial inflammation and proliferation, concurrent with the generation of autoantibodies, culminating in the deterioration of cartilage and bone, and consequent disability 1. It is hypothesized that the onset and progression of RA result from a multifaceted interplay of genetic, environmental, and life style determinants 2,3. This condition preferentially afflicts the geriatric female demographic, accounting for approximately 1% of the global population. Manifesting primarily through inflammation in small and medium-sized joints, RA substantially erodes the quality of patients' life 4,5. Further to this, sustained inflammation poses risks to additional organs, including the cardiovascular system and kidneys. Despite this, extant therapeutic strategies largely center on ameliorating inflammatory symptoms rather than providing comprehensive RA mitigation, and are frequently associated with a spectrum of adverse effects 6.

Contemporary understanding posits that the pathogenesis of bone and cartilage erosion in RA is predominantly due to the interactions involving both the innate and adaptive immune cohorts. Noteworthy is the aberrant activation of macrophages, innate immune cells, during the incipient phases of the disease 7. RA-afflicted synovia witness a pronounced influx of these macrophages, believed to be sourced from both circulating monocytes and sessile tissue-resident populations. Monocyte-derived macrophages are progeny of hematopoietic stem cells located in the bone marrow, whereas resident macrophages trace their lineage back to embryonic development stages in the fetal liver and yolk sac. The phenotypic plasticity of macrophages is considerable, allowing them to adapt their functional stance in response to the evolving extracellular milieu 8. Upon environmental cueing, they shift towards a pro-inflammatory stance, unleashing a cascade of inflammatory cytokines, including IL-1β, IL-12, IL-6, TNF-α and chemokines, which collectively conscript additional immune effector T and B cells to the site, thus exacerbating the inflammatory milieu within the joint space 9.

Pyroptosis, a lytic and inflammatory form of programmed cell death, primarily documented in relation to infectious disease pathologies, has recently garnered attention for its role in autoimmune diseases, including RA 10,11. Investigations have underscored the occurrence of macrophage pyroptosis in RA, implicating it as a driver in the disease's pathogenesis. This cell death pathway is distinguished by a swift breach of the cell membrane accompanied by the discharge of inflammatory mediators, most notably IL-1β and IL-18 12. The process is initiated when macrophages recognize exogenous danger cues such as lipopolysaccharide (LPS). This detection precipitates the assembly of the NLRP3 inflammasome, a multiprotein complex involving the NOD-like receptor thermal protein domain associated protein (NLRP3), the apoptosis-associated speck-like protein containing a caspase recruitment domain (ASC), and procaspase-1. Subsequent activation triggers the release of IL-1β and IL-18 following their cleavage by caspase-1. Beyond eliciting an inflammatory response, macrophage pyroptosis fosters the aberrant clonal expansion of fibroblast-like synoviocytes and the secretion of matrix metalloproteinases, thus magnifying tissue damage and promoting irreversible bone erosion in RA 13,14.

Salt is a common seasoning that enhances the flavor of food. The World Health Organization recommends that sodium chloride intake should be less than 5 grams per person per day 15. However, statistics indicate that the per capita salt intake in most countries is between 9-12 grams or even higher, far exceeding the recommended amount. Excessive salt intake not only increases the risk of hypertension and cardiovascular disease, but recent studies have also demonstrated that a high-salt diet can lead to abnormal changes in the immune system, thereby increasing the risk of multiple sclerosis and systemic lupus erythematosus 16.

The impact of high-salt diet on the progression of RA is still inconclusive. A clinical study from Spain indicated that individuals with high sodium intake had a 50% increased risk of developing RA 17. In this study, we have engineered a murine model to investigate the influence of dietary salt on collagen-induced arthritis (CIA) by employing DBA/1 mice. The model consisted of administering a high-salt diet to evaluate its impact on joint inflammation. Our observations reveal that mice fed with such a diet exhibited exacerbated joint destruction in the context of CIA. Further molecular investigations disclosed an upregulation of serum and glucocorticoid-regulated kinase 1 (SGK1) activity in response to elevated NaCl levels. This upregulation appeared to precipitate the activation of the mitogen-activated protein kinase (MAPK) p38 signaling pathway, which, in turn, potentiated the pyroptotic death of macrophages, thereby intensifying joint inflammation. These findings underscore the potential peril associated with high salt consumption as an aggravating factor for RA. Consequently, we propose adopting a low-salt diet as a strategic measure for individuals contending with RA. Additionally, our results nominate SGK1 as a promising molecular target for the development of innovative RA therapeutic strategies.

## Materials and Methods

### Clinical data

The clinical data were sourced from patients admitted to the rheumatology department of the Second People's Hospital of Hefei between January 2022 and April 2023. The majority of patients included were aged between 35 and 85 years old (n=100), with a diagnosis of either RA or osteoarthritis (OA). Patients with a history of hypertension or heart disease, and those who required treatment with drugs containing sodium or potassium, were excluded from the study. Patients with RA were diagnosed using the 2010 classification criteria established by the American College of Rheumatology and the Federation of European Societies of Rheumatology. The diagnostic criteria for OA were in accordance with the Bone and Joint Diagnosis and Treatment Guidelines updated by the Orthopaedic Branch of the Chinese Medical Association in 2018.

### Specimens

In this study, we utilized human knee synovium tissues from RA (n = 5) and OA (n = 5) patients undergoing total joint replacement in the Department of Orthopaedics of Hefei Second People's Hospital. All patients provided written informed consent prior to the experiment, and the study protocol was approved by the Biomedical Ethics Committee of Anhui Medical University (Approval No. 83230267).

### Animals

Seven to eight-week-old male DBA/1 mice (average weight 18±2g) were procured from Gem Pharmatec (Nanjing, China) and were maintained in a Specific Pathogen Free (SPF) animal house, under conditions of 25°C and a 12-hour light-dark cycle. In the experiment, cage bias was taken into consideration. Instead of being fed in a separate ventilated cage, they were all kept in the same open environment. This study was approved by the Experimental Animal Ethics Committee of Anhui Medical University (Approval No. 20220454).

### HSD treatment

The mice were randomly assigned to either the NSD (Normal mice fed standard diet and provided regular drinking water) or HSD (High-salt diet containing 4% added NaCl and drinking water with 1% NaCl) groups 18, with 20 mice in each group. Prior to the experiment, the mice in the HSD group were acclimated to the high-salt diet for one week. After seven days, ten mice from each group were randomly selected to establish the collagen-induced arthritis (CIA) model. The mice were then divided into four experimental groups, with ten mice per group: NSD + normal group (NSD), HSD + normal group (HSD), NSD + CIA model group (NSD + CIA), and HSD + CIA model group (HSD+CIA). Throughout the duration of the experiment, the body weight, dietary intake, and water consumption of each group were measured at two-day intervals.

### CIA mouse model

Fully emulsified chicken type II collagen (Chondrex, cat#20011, USA) was combined with complete Freund's adjuvant (Chondrex, cat#7023, USA) containing 5 mg/mL inactivated Mycobacterium tuberculosis. On day 0, 8-week-old male DBA/1 mice received a subcutaneous injection of 100 μL of the emulsified collagen at the tail root. On day 21, a second injection of 100 μL was administered to enhance immunity. The clinical severity of arthritis was evaluated between 29 and 49 days after the initial immunization.

### Evaluation of arthritis

To assess the severity of CIA, two independent observers, unaware of the experimental protocol, conducted a comprehensive evaluation. Beginning on the 29th day after the initial immunization, the mice's clinical scores were measured three times every two days, including arthritis index, paw swelling number, and body weight. The criteria for arthritis index scoring were as follows: 0 = normal; 1 = erythema and slight swelling of the ankle joint; 2 = erythema and slight swelling from ankle to metatarsal or metacarpal joints; 3 = erythema and moderate swelling of ankle to metatarsophalangeal or metacarpal joints; 4 = erythema and severe swelling of ankle to toe joints. The paw swelling number of mice in each group was also recorded every 2 days from day 0, with each mouse's paw consisting of 1 ankle joint and 5 knuckles. A single spot of redness was scored as 1 point, with each mouse having a maximum score of 24.

### Histological analysis of joint and spleen

To ascertain the histopathological hallmarks, we employed Hematoxylin and Eosin (H&E) staining. Hind limbs and spleens excised from the subjects were initially preserved in 4% paraformaldehyde for a duration of 24 hours. After the fixation process, hind limbs underwent a decalcification protocol for 30 days, with the decalcifying solution replenished biweekly. In contrast, spleens were promptly processed for paraffin embedding. We assessed inflammatory manifestations and the extent of bone erosion within the knee and ankle joints by adopting a systematic approach that incorporated an analysis of synovial hypertrophy, infiltration of inflammatory cells, pannus formation, and the integrity of bone and cartilage. This assessment was performed independently by a duo of experienced investigators to ensure objectivity. In addition to the joint evaluation, a separate pair of independent researchers conducted H&E staining on spleen tissue samples, whereby they quantified alterations based on parameters which included changes in spleen morphology, the prevalence of germinal centers, and the degree of leukocytic infiltration.

### Safranin O/Fast green staining

Following the removal of wax, tissue sections were immersed in safranin O for three minutes, then briefly rinsed in distilled water for one minute. Fast green staining ensued for two minutes, succeeded by another distilled water rinse. Differentiation was achieved with a one-minute treatment in 1% acetic acid. Progressive dehydration was conducted using 95% ethanol, followed by clearance in xylene. The morphological alterations of the samples were then examined post-mounting with neutral balsam.

### Mouse primary peritoneal macrophages isolation and culture

Following euthanasia by cervical dislocation, the corpses of mice were sanitized through a five-minute submersion in 75% ethanol. The abdominal integument was retracted to reveal the peritoneal cavity. A cooled phosphate-buffered saline (PBS) solution was injected into the splenic cavity, succeeded by gentle abdominal massaging for five minutes to dislodge immune cells. Peritoneal exudate was then aspirated using a syringe armed with a 21-gauge needle. The resultant fluid was centrifuged at 4°C at 400×g for ten minutes to precipitate macrophages. These cells were then re-suspended in culture medium and enumerated. The macrophages were seeded in Roswell Park Memorial Institute (RPMI) 1640 medium, procured from VivaCell in Shanghai, enhanced with 10% fetal bovine serum from WISENT, based in Nanjing, and buffered with an antibiotic-antimycotic cocktail comprising 1% penicillin and 1% streptomycin, both sourced from Beyotime in Shanghai. Following adherence to culture vessels, the cells were appropriated for experimental evaluations.

### Cell culture and treatment

RAW264.7 cell line (Procell Life Science & Technology Co., Ltd, Wuhan, China) in 10% fetal bovine serum (WISENT, China was cultured in medium (High sugar DMEM) (VivaCell, Shanghai, China) and placed in an incubator at 37°C and 5% CO_2_. Mononuclear leukemia cell line (THP-1 cells) (Procell Life Science & Technology Co., Ltd, Wuhan, China) in 15% fetal bovine serum (WISENT, Nanjing, China) was cultured in medium (RPMI 1640) (VivaCell, Shanghai, China) and placed in an incubator at 37°C and 5% CO_2_. Prior to the experiment, THP-1 monocytes were transformed into M0 adherents after being treated with 100 nM phorbol12-myristate 13-acetate (PMA) (GLPBIO, CA, USA) on a 6-well plate for 48h. LPS (1μg/mL) (Solarbio, Beijing, China) in combination with ATP(5μM) (Sigma, USA) induces pyroptosis in Raw264.7 cells, whereas in THP-1-derived macrophages, pyroptosis is induced using only LPS. Slc6a12 inhibitor (BPDBA) (MCE, Nj, USA) was added to the six-well plate with a final concentration of 20μM, and the cells were stimulated for 24h. NaCl pretreatment was 50 mM for 12h.

### Western blotting (WB)

The total protein was separated by 10% fast polyacrylamide (PAGE) gel (YEASEN, Shanghai, China), and the isolated protein was transferred to PVDF membrane (Millipore, MA USA). Sealed with 5% skim milk at room temperature for 2h, then with specific primary antibody at 4°C. Rabbit anti-β-actin (Cat#: AF7018, Affinity) and rabbit anti-phospho-SGK1 (Ser422) (Cat#: AF3001, Affinity) were purchased from Affinity Biosciences (Cincinnati, OH, USA). Rabbit anti-phospho-p38 MAPK (Thr180/Tyr182) (Cat#: 4511) and rabbit anti-p38 MAPK (Cat#: 8690) antibodies were purchased from Cell signaling technology (MA, USA). Anti-NF-κB p65 recombinant rabbit monoclonal antibody (Cat#: ET1603-12), anti-phospho-NF-κB p65(S529) recombinant rabbit monoclonal antibody (Cat#: ET1604-27) and anti-Gasdermin D (N terminal) recombinant rabbit monoclonal antibody (Cat#: HA721144) were purchased from HUABIO Co., LTD (Hangzhou, China). Rabbit anti-IL-18 (Cat#: R24693) and rabbit anti-cleaved-Caspase-1 (Cat#: 341030) antibodies were purchased from ZEN-BIOSCIENCE (Chengdu, China). Rabbit anti-IL-1beta (Cat#: 66737-1-Ig), rabbit anti-SGK1 (Cat#: 28454-1-AP) and mouse anti-Slc6a12 (Cat#: 67700-1-Ig) antibodies were purchased from Proteintech group (Wuhan, China). Rabbit anti-NLRP3 (Cat#: WL02635) antibody was purchased by WANLEIBIO (Shenyang, China). After washing with PBS for 3 times, it was incubated with secondary antibody (Elabscience, Wuhan, China) at room temperature for 2h. After washing with TPBS, bands were observed with enhanced chemiluminescence reagent (Affinity, USA) by fully automated chemiluminescence imaging system (Tanon 5200, China).

### Immunohistochemical staining

Mice was sacrificed on Day49, the knee joints were fixed and decalcified, dehydrated with fractional alcohol, embedded in paraffin, and sectioned for 5µm. The tissue sections were placed in an oven at 55°C overnight and immersed in xylene dewaxing and gradient ethanol. Washed with PBS 3 times for 15 minutes, washed with PBS 3 times, the slices were boiled in a large antigen repair solution at 100°C and cooled to room temperature. Wash with PBS 3 times, seal with endogenous peroxidase for 10 minutes, rinse with PBS 3 times, seal and serum for 15 minutes. The primary antibody F4/80 (Cell signaling technology, MA, USA) was then incubated overnight at 4°C. The next day, biotin-labeled secondary antibody was incubated at room temperature for 20 minutes, washed with PBS for 3 times, labeled with horseradish peroxidase marker, washed with PBS for 3 times, colored with DAB, stained with hematoxylin, dehydrated with gradient alcohol, and mounted with neutral adhesive. Finally, the tissue staining results were observed in the digital slice scanning system Pannoramic MIDI (Pannoramic MIDI II, Hungary, China).

### Immunofluorescence staining

The RAW264.7 cells were adhered to a 24-well plate with a 1.5 mm polylysine coating. After pretreatment with NaCl (50 mM) for 12 hours, the RAW264.7 cells were stimulated with LPS and ATP. After 24 hours, the cells were fixed with acetone for 15 minutes at room temperature, followed by infiltration with PBS + 0.1% Triton X-100 for 15 minutes. Subsequently, the cells were washed three times with PBS and blocked with 5% BSA at room temperature for 1 hour to prevent non-specific antibody binding. Primary antibody staining was carried out overnight using NLRP3 antibody (HUABIO, Hangzhou, China) dissolved in 5% BSA at a 1:200 ratio. A secondary antibody conjugated with Alexa Fluor 488 (715-545-151, Jackson Immunol Research) was applied according to the manufacturer's instructions. The secondary antibody was incubated with the cells for 1 hour, followed by three washes with PBS. The cells were then incubated with DAPI for 10 minutes, washed three times to remove water stains, and finally, an anti-fluorescence quencher was applied to seal the slide cover. Images were obtained using a laser scanning confocal microscope (Leica SP8, Germany) and saved for analysis.

### Scanning electron microscopy

The RAW264.7 cells were seeded onto a 1.5 mm polylysine coated surface of a 24-well plate. Consistent with previous studies 19, Prior to inducing pyroptosis, RAW264.7 cells were pretreated with NaCl (50 mM) for 12 hours. After 24 hours, the cells were fixed in 2% glutaraldehyde solution (prepared in PBS) for 2 hours. Subsequently, the samples were dehydrated using a series of ethanol gradients ranging from 20% to 100% (20%, 40%, 60%, 80%, and 100%). Each ethanol concentration was maintained for approximately 30 minutes. Following dehydration, the samples were subjected to vacuum-assisted drying with 100% acetone, followed by critical point drying. Finally, the samples were gold-plated and observed under a GeminiSEM300 (Zeiss, Germany) scanning electron microscope.

### Flow cytometry

Mouse paws were sectioned into 3-4 mm tissue pieces and placed in 1.5mL EP tubes. Hank's equilibrium salt solution (1mL), type II collagenase (1g/mL, 2μL), and CaCl2 solution (3mM, 5μL) were added. The mixture was then incubated at 37°C for 4 hours. After centrifugation and three washes with PBS, the cells were obtained through nylon sieving to yield a dispersed cell suspension. The treated single-cell suspension (1×10^6^/100 μL) was incubated in the dark at 4°C for 30 minutes with a fluorescent antibody. Subsequently, the cells were centrifuged at 300 g, washed three times with PBS, and resuspended in 300 μL of PBS for cell sorting observation. The isolated cells were stained with the following antibodies: CD45-FITC (553080, BD Pharmingen), CD19-PE (553786, BD Pharmingen), CD3-PE (553063, BD Pharmingen), CD4-FITC (553650, BD Pharmingen), CD11b-FITC (101206, biolgend), Ly6G-PE (551461, BD Pharmingen), and Ly6C-APC (553129, BD Pharmingen).

### ELISA for cytokine measurements

RAW264.7 macrophages were seeded in 6-well plates at a density of 5×10^5^ cells/mL. Prior to inducing pyroptosis, the cells were pre-incubated with NaCl (50 mM) for 12 hours. Subsequently, the cell culture supernatant was centrifuged at 4°C and 1500 rpm for 15 minutes to discard any particles. The levels of secreted IL-1β and IL-18 were quantified using a commercially available mouse IL-1β and IL-18 ELISA kit (MLBIO, Shanghai, China), following the manufacturer's instructions. For measurement, mouse serum samples were appropriately diluted.

### Quantitative real-time PCR (RT-qPCR)

Total RNA was extracted from mouse paw tissues and cells using TRIzol. A 20 μL total system of 5× reverse transcriptase was used for cDNA synthesis. The cDNA was subsequently diluted four times, and PCR amplification was conducted according to the manufacturer's instructions. The PCR reaction volume was set to 20 μL, and the amplification conditions were as follows: initial denaturation at 95°C for 5 minutes, followed by 40 cycles of denaturation at 95°C for 10 seconds, annealing at 60°C for 35 seconds, and extension at 72°C for 15 seconds. A melting curve analysis was performed by heating the samples to 95°C for 15 seconds, cooling to 60°C for 60 seconds, and then heating again to 95°C for 15 seconds. The expression of target genes was quantitatively analyzed using the 2-^ΔΔCT^ method, with β-actin or GAPDH genes used as internal references. The primer sequences for RT-qPCR are provided in Table 1.

### Lactate dehydrogenase (LDH) based cytotoxicity assay

*In vitro*, pyroptosis was assessed using the lactate dehydrogenase cytotoxicity assay kit (Beyotime, Shanghai, China). RAW264.7 cells were seeded in 96-well plates, and following the addition of the desired stimulus, the cell supernatant was collected as per the manufacturer's instructions for subsequent detection.

### PI staining

The RAW264.7 cells were seeded onto a 1.5 mm polylysine-coated surface of a 24-well plate. Following a 12-hour pretreatment with NaCl (50 mM), the RAW264.7 cells were subsequently stimulated with LPS and ATP. After a 24-hour incubation period, the cells were stained using the (Beyotime, China) assay kit, according to the supplier's instructions. Necrotic cells were labeled with propidium iodide (PI), while living cell nuclei were labeled with Hoechst33342. Subsequently, images were acquired and analyzed using a Leica fluorescence microscope, after sealing the samples with a fluorescence quencher (Solarbio, China).

### RNA‑sequencing and data analysis (RNA-seq)

RNA sequencing and subsequent data analysis were conducted to examine the transcriptomic profiles of RAW264.7 cells. RNA extraction was performed using an RNA sequencing kit (APEBIO, Shanghai, China). Differential gene expression was analyzed using either DESeq2 or EdgeR, comparing biological duplicate samples within the same group or without a biological duplicate sample within the group, respectively. Volcano plots were used to display up-regulated and down-regulated differential genes. The screening criteria for differential genes were set as |log2FC| ≥ 1 and p-adjust ≤ 0.05. To explore the functional implications of the differentially expressed genes, Kyoto Encyclopedia of Genes and Genomes (KEGG) and Gene Ontology (GO) enrichment analyses were carried out using the R-package ClusterProfiler (v4.2.2). Visualization of genomic data was achieved using heat maps and hierarchical clustering techniques. Significantly enriched pathways were identified based on a screening criterion of P ≤ 0.05, indicating the overexpression of known biological functions or processes in the list of experimentally obtained differences. Gene Set Enrichment Analysis (GSEA) was employed to sort all genes according to log2FC values, ranging from small to large. Meaningful gene sets were determined using the criteria |NES| > 1, P < 0.05, and FDR/q-val < 0.25, reflecting the extent of enrichment and statistical significance.

### Lentivirus transduction and construction of SGK1 knock down cell line

To construct an SGK1-silenced THP-1 cell line, THP-1 cells were infected with lentiviral particles. Lentivirus expression vector LV-Control, LV-shSGK1 was purchased from Anhui General Biology Co., LTD (sequense: GGATGGGTCTGAA CGACTTTA) 20. Simply put, before lentiviral particle infection, the cells were inoculated on a 6-well plate at a rate of 2×10^5^ cells per well, incubated with 2 mL complete medium for 24h, and then the lentiviral particles were infected with the appropriate MOI value for screening, and 5 μg/mL polybrene was added, gently shaken, and cultured at 37°C overnight. 24h later, the cells in each well were collected into a sterile 1.5mL EP tube, centrifuged at 250g for 3min, the super serum was removed, replaced with a complete medium, gently mixed, and then placed back into the culture plate for further culture at 37°C. The infected cells were then screened with 2mg/mL of puromycin for 96h, and the dead cells without successful infection were discarded and the blank lentiviral vector was used as the control. After screening, fluorescence microscopy, RT-qPCR and western blot were used for verification.

### THP-1 cells were transfected with overexpression plasmids

SGK1 knockout THP-1 cells in the logarithmic growth phase were inoculated into a 6-well plate (4×10^5^ cells per well, total volume 2mL). They were induced into macrophages by PMA (100 nM) for 48h. The experiment was carried out according to the DNA transfection protocol of Lipofisher transfection reagent (HUABIO, Hangzhou, China): taking one well as an example, 2.5 μg DNA was diluted in 125 μL base medium and mixed for 2 min, and 5 μL transfection reagent was diluted in 125 μL base medium and mixed for 2 min. Then the two were mixed for 20 min and slowly dropped into the hole and placed in the incubator for culture. After 6-8h, the culture medium was replaced with complete culture medium. After transfection for 48 h, RT-qPCR and Western blot were used for verification.

### Measurements of intracellular Na^+^ flux

SoNa 520 AM was measure to detect transient changes in intracellular Na^+^ levels. 100 μL of THP-1 cells (4×10^5^ cell/mL) were seeded in a 96-well plate, washed 3 times with calcium-free buffer, and then incubated with 5 μM SoNa 520 AM and 0.04% Pluronic F127 for 1 h to bind sodium ions. After standing for 1 h, the cells were washed with calcium-free buffer 3 times to remove excess dye. Subsequently, the concentration of intracellular Na^+^ indicating ion flux was measured with the microplate reader CYTATION-5 (BioTek, VT, USA), which measured the fluorescence emitted every 10 s for a total of 400 s. The cytosolic Na^+^ concentration level was determined on the basis of the SoNa 520 AM fluorescence emitted at both 490 and 525 nm (the ratio of 490 nm/525 nm). At 270s, NaCl (50 mM) was added, and then, the changes to the intracellular Na^+^ concentration was measured.

### Statistics and reproducibility

Statistical analysis was performed using PRISM V.9.0.0 (GraphPad, USA) software. The data in the figure are expressed as Mean ± SEM. For statistical comparison, T test was used for comparison between two groups or paired comparison; for comparison between two or more groups under the same conditions, Tukey post test was used after one-way ANOVA; for comparison between two or more groups under two conditions, Sidak's or Tukey's post test was used after two-factor ANOVA. p<0.05 was considered significant. For all statistical analyses of normalized data, the logarithm of each data point is calculated (on a 2 basis) and these converted values are used for statistical analysis.

## Results

### High-Salt diet exacerbates CIA in mice

**Figure 1:**
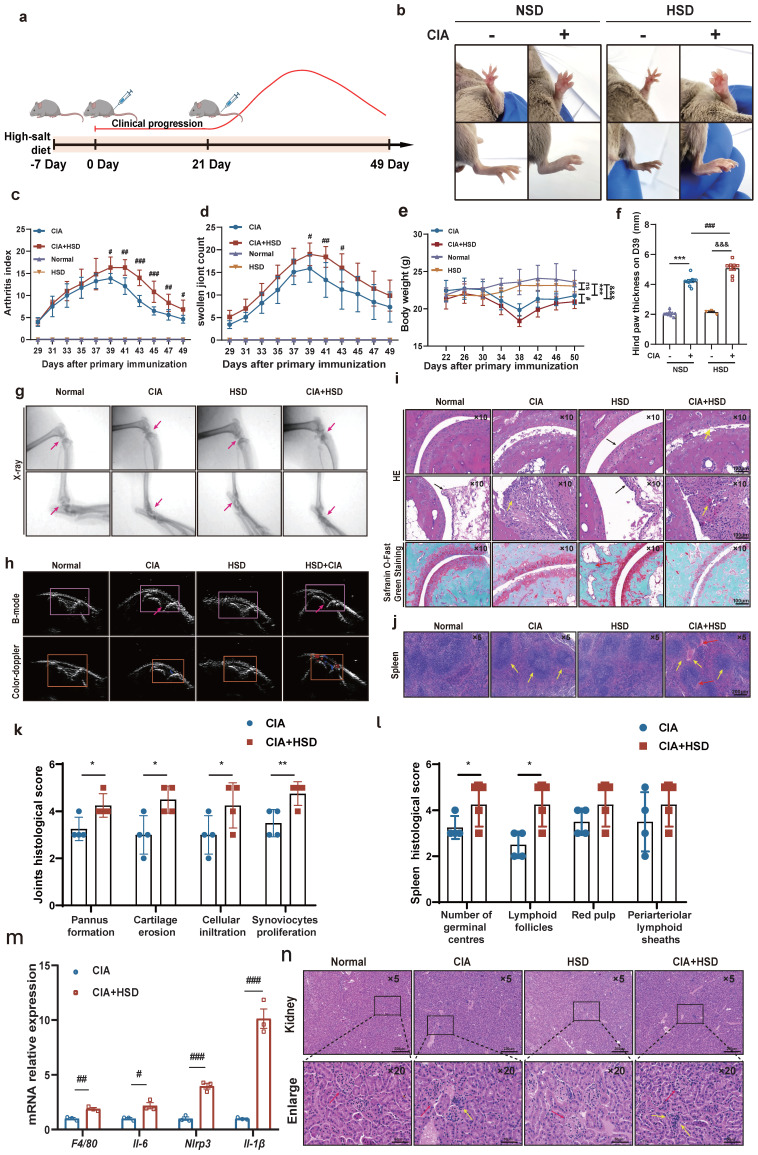
** HSD exacerbates the severity of disease in CIA mice. (a)** Flowchart of CIA mouse model establishment and HSD treatment.** (b)** The representative photos of the feet of each group of mice on the day 39. Graphs of arthritis index **(c)**, number of swollen paws** (d)** and body weight **(e)** of mice in each group over time. Data are expressed as the mean ± SD (n=10). *#p*<0.05, *##p*<0.01, *###p*<0.001, HSD+CIA group *vs* CIA group.* &&&p*<0.001 HSD+CIA group *vs* HSD group. ***p<0.001 CIA group *vs* Normal group. **(f)** Paw thickness on day D39 in each group of mice. **(g)** X-ray images of knee and ankle joints of each group of mice (Diagonal upward red arrow indicated clear articular surfaces and smooth articular cavities in the NSD and HSD groups, while diagonal downward red arrow indicated narrow interarticular and bone injury in the CIA groups). **(h)** Ultrasonic images of the knee joints in B-mode and by color Doppler (The yellow arrow marks the dark fluid in the cavity, which can reflect the expansion of the fluid). **(i)** Representative pathological images of H&E and Safranin O/Fast green staining of ankle joint (Scale Bar: 100μm) (Black arrow indicates that there is no inflammatory cell infiltration and synovial hyperplasia in normal and HSD mice, and the cartilage surface is smooth and complete. Yellow arrow refers to synovial hyperplasia and inflammatory cell infiltration in two groups of CIA mice). **(j)** H&E images of spleen of each mice group (Scale Bar: 200μm) (Yellow arrow indicates germinal center and lymphatic follicle, red arrow indicates red pulp congestion). Statistical chart of joints** (k)** and spleen** (l)** pathology scores. Data are expressed as the mean ± SD (n=4). *p<0.05, **p<0.01. **(m)** Relative expression of mRNA of *F4/80*, *Il-6*, *Nlrp3*, *Il-1β* in the paws of four groups of mice. Data are expressed as the mean ± SD.* #p*<0.05, *##p*<0.01, *###p*<0.001. (n) Representative images of H&E staining of mouse kidneys from each group (Scale Bar: 200 μm or 50 μm) (Red arrows indicate the glomerular structure, yellow arrows indicate infiltrating inflammatory cells).

To explore the impact of a high-salt diet (HSD) on arthritis progression, we induced CIA in DBA/1 mice by subcutaneously injecting chicken type II collagen on days 0 and 21. These mice were divided into NSD and HSD group. One week prior to immunization, the HSD-fed mice received a 4% NaCl diet and 1% NaCl drinking water (Fig. **1**a).

Throughout different stages of inflammation, we recorded the arthritis index, foot swelling, body weight, and water and diet intake for the four groups of mice every 2 days. At approximately 29 days after the initial immunization, both NSD and HSD-fed CIA mice began to experience paw swelling, reaching peak inflammation around day 39. Importantly, the CIA mice fed an HSD exhibited more severe swelling in their front and back paws and ankles compared to those on NSD (Fig. **1**b). Both the NSD and HSD-fed CIA mice exhibited peak arthritis index and foot swelling numbers at around day 39, signifying significant weight loss. However, the HSD-fed CIA group displayed significantly higher arthritis index, paw swelling numbers, prolonged peak inflammation duration, delayed resolution, and increased weight loss compared to the NSD-fed CIA group (Fig. **1**c-e). Meanwhile, we used vernier caliper strategy to measure the foot thickness of mice on D39, results also showed that the longitudinal distance of the posterior paw in HSD-fed CIA mice was larger (Fig. **1**f).

X-ray observations revealed that the articular cavity in control mice on both NSD and HSD appeared clear, with smooth joint surfaces (Red arrow pointing diagonally upwards). Conversely, both CIA groups displayed joint space narrowing and bone injury, with HSD-fed CIA mice exhibiting even narrower joint cavities, more blurred bone boundaries, and more pronounced bone injuries (Red arrow pointing diagonally downwards) (Fig. **1**g). Ultrasound imaging revealed the articular cavity as an inverted triangle structure formed between the femur, tibia, and patellar ligaments of the knee joint. The relative area of dark fluid within this cavity indicated fluid accumulation (Red arrow). Both the NSD and HSD-fed CIA mice exhibited significantly increased dark areas in the joint cavity compared to their respective control group mice. However, HSD-fed CIA mice displayed even larger dark areas within the joint cavity, indicating more severe inflammatory joint effusion. Color Doppler blood flow signals demonstrated that intra-articular blood flow signals in both CIA groups were significantly stronger than in their respective controls, with the HSD-fed CIA mice exhibiting stronger color patterns (Fig. **1**h).

Hematoxylin and eosin (H&E) staining of ankle joints showed that the synovium in normal and HSD-fed mice was single or double-layered, without inflammatory cell infiltration or synovial hyperplasia. The cartilage surface remained smooth and intact (black arrow).

In contrast, both CIA groups showed more pronounced cartilage injuries, synovial hyperplasia, and inflammatory cell infiltration (yellow arrow). However, HSD-fed CIA mice exhibited a narrower joint space and more evident pannus formation (Fig. **1**i and k). Similarly, Safranin O/Fast green staining revealed that the cartilage in control mice, whether on NSD or HSD, was darker red, smoother, and intact. In contrast, CIA mice on both diets exhibited lighter red cartilage, eroded cartilage surfaces, and uneven staining. Notably, HSD-fed CIA mice showed even lighter cartilage staining and smaller cartilage thickness, indicating more severe cartilage erosion (Fig. **1**i). H&E staining of spleen sections showed that compared to normal mice on both NSD and HSD, CIA mice displayed more germinal centers and lymphatic follicles (red arrow). Furthermore, HSD-fed CIA mice exhibited even more germinal centers (yellow arrow) compared to CIA mice fed-on NSD (Fig. **1**j). RT-qPCR analysis of whole paw homogenates revealed that HSD feeding significantly increased mRNA levels of *Il-6*, *Nlrp3*, *Il-1β*, and *F4/80* (the mouse macrophage marker) in the joints of CIA mice (Fig. **1**m). This suggests that a high-salt diet aggravates joint damage may be related to macrophages. Considering that high sodium chloride intake may cause kidney damage, we investigated whether HSD affected the kidneys of the animals and observed no significant difference in H&E staining between the kidneys of mice in the NSD and HSD groups. However, inflammatory cell infiltration was detected in the kidneys of CIA mice on both NSD and HSD (Fig. **1**n) 21.

In summary, these findings collectively demonstrate that HSD markedly exacerbates clinical manifestations in CIA mice, resulting in more severe joint inflammation and cartilage injury.

### HSD enhances macrophage infiltration and promotes transition to inflammatory phenotypes in CIA mice

**Figure 2:**
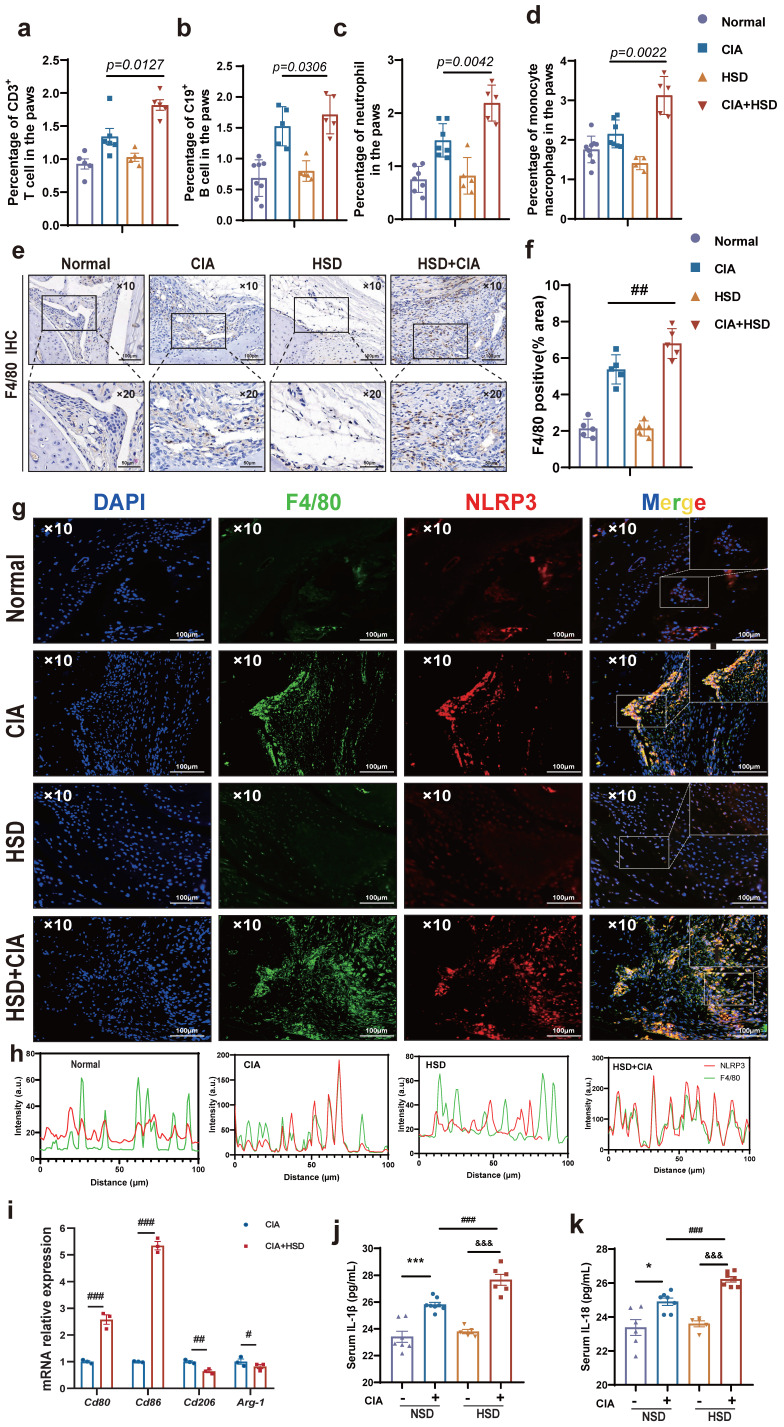
** HSD increases macrophage infiltration and inflammatory lesions in the CIA mice.** The proportion of CD3^+^ T cell **(a)**, CD19^+^ B cells **(b)**, CD11b^+^Ly6G^+^ neutrophils **(c)**, CD11b^+^Ly6C^+^ monocytes and macrophages **(d)** in mice paws detected by flow cytometry. Data are expressed as the mean ± SD (n=5-8). **(e)** Representative immunohistochemical image of macrophage marker F4/80 aggregation in joint tissue (Scale Bar: 100μm and 50μm). **(f)** Statistical chart of F4/80 positive areas. **(g)** The expression and colocalization of F4/80 and NLRP3 in the joint tissues of mice in each group were analyzed by immunofluorescent staining (DAPI blue, F4/80 green, NLRP3 red) (Scale Bar: 50μm). **(h)** F4/80 and NLRP3 co-localization statistic graph, the HSD + CIA group has stronger fluorescence intensity and co-localization.** (i)** Relative expression levels of mRNA of M1 macrophage marker gene *Cd80*, *Cd86* and M2 macrophage marker gene *Cd206* and *Arg-1* in peritoneal macrophages of four groups of mice. Data are expressed as the mean ± SD.* #p*<0.05, *##p*<0.01, *###p*<0.001. **(j-k)** The level of IL-1β and IL-18 in mouse serum were examined by ELISA (n=6-8).

In order to probe into the specific mechanism of HSD aggravating CIA, we first used flow cytometry to detect the proportion of various immune cells in mice paws to explore the main immune cell groups mainly affected by HSD. The results showed that the number of T cells (CD3^+^), B cell (CD19^+^), neutrophils (CD11b^+^Ly6G^+^), and mononuclear macrophages (CD11b^+^Ly6C^+^) in paws of CIA mice fed with both NSD and HSD were significantly increased compared with the control group (Fig.**2**a-d, sFig **1**). Importantly, HSD-fed CIA mice exhibited a significantly higher proportion of various immune cells in their paws compared to NSD-fed CIA mice, with the most substantial increase observed in mononuclear macrophages (*P*=0.0022) (Fig. **2**d), which was consistent with the significant upregulation of F4/80 mRNA levels in the paw tissues of mice. Immunohistochemical images also showed a large F4/80 labeled macrophage infiltration in the ankle tissues of CIA mice compared to the control group with a normal diet (Fig. **2e**). As expected, more macrophages accumulated in the joints of the HSD-fed CIA mice compared to the NSD arthritic mice (Fig. **2**f). Pyroptosis is the most common form of macrophage exacerbation validation, so we examined the expression of NLRP3 in macrophages of mice in each group. Immunofluorescence staining further demonstrated a significant increase in F4/80-positive macrophages and co-expression of F4/80 and NLRP3 in ankle tissues of CIA mice compared to the control group on a normal diet (Fig. **2**g). Moreover, when compared to NSD-fed CIA mice, the number of F4/80-positive macrophages and F4/80 and NLRP3 double-positive cells in ankle tissues of HSD-fed CIA mice was significantly higher within the same area (Fig. **2**h). Collectively, these findings indicate that HSD influences the function of macrophages within the joint tissues of CIA mice.

To further explore the effects of HSD on macrophages, peritoneal macrophages from mice in each group were isolated for RT-qPCR analysis. The results demonstrated that the mRNA levels of *Cd80* and *Cd86*, markers associated with M1 macrophages, were significantly increased in the abdominal macrophages of CIA mice on an HSD when compared to those on NSD. In contrast, mRNA levels of *Cd206* and *Arg-1*, markers linked to M2-type macrophages, were significantly decreased (Fig. **2**i). Additionally, ELISA results indicated that the expression levels of IL-18 and IL-1β in the serum of CIA mice were significantly elevated, regardless of their diet. However, the expression levels of these cytokines in the serum of HSD-fed CIA mice were notably higher (Fig. **2**j-k).

**Figure 3:**
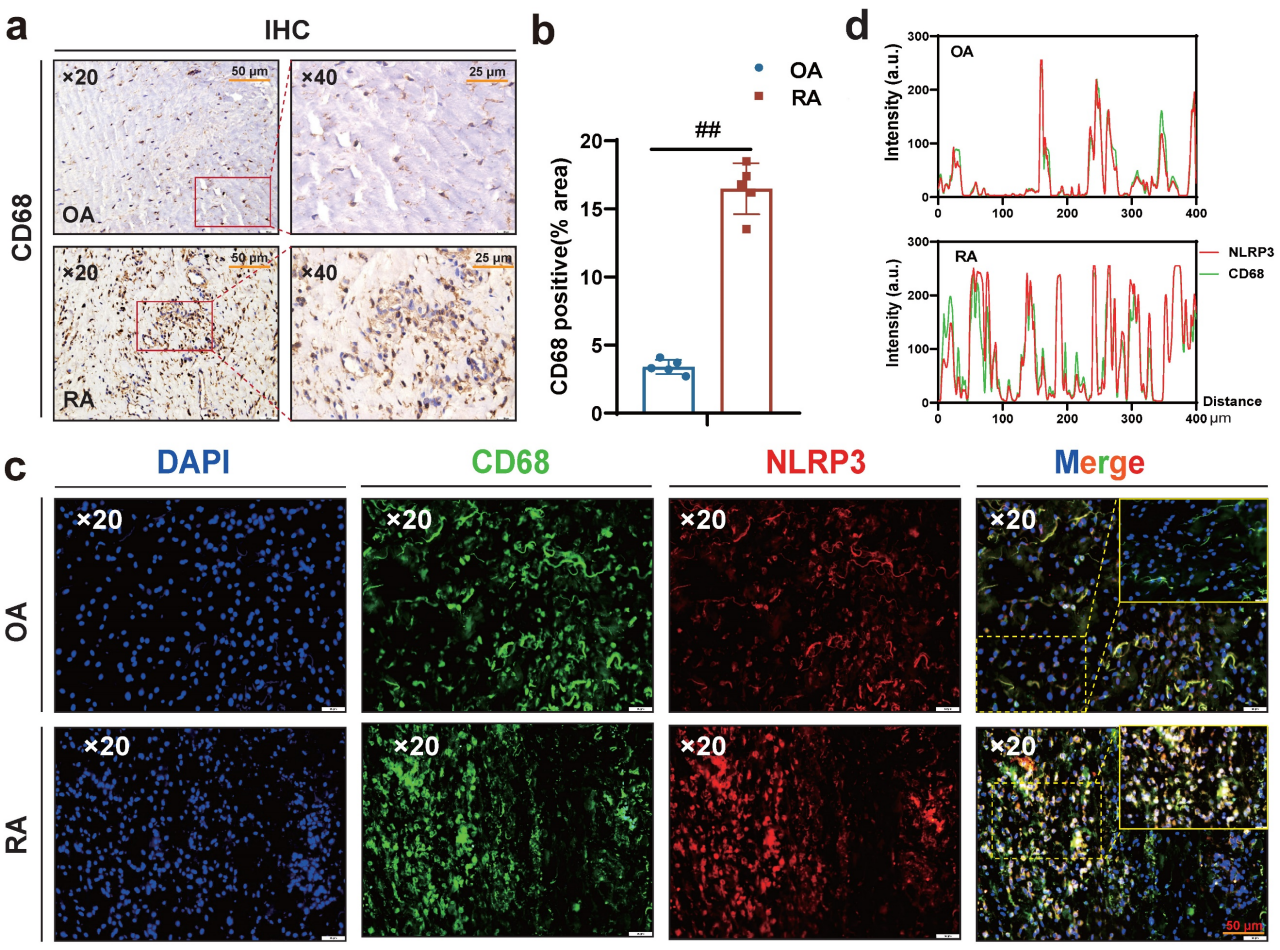
** A large number of CD68-positive macrophages undergo pyroptosis in RA synovial tissue. (a)** Representative images showing human macrophage marker CD68 expression in the synovium of OA and RA patients by IHC (Scale Bar: 50 μm and 25 μm). **(b)** Statistical chart of CD68 positive areas (n=5). **(c)** CD68 and NLRP3 expression and co-localization in the synovium of OA and RA patients were detected by immunofluorescent staining (DAPI blue, CD68 green, NLRP3 red) (Scale Bar: 50μm). **(d)** CD68 and NLRP3 co-localization statistic graph.

Interestingly, macrophages were labeled with CD68, and immunohistochemical results showed more macrophage infiltration in RA synovium (Fig. **3** a-b), while immunofluorescence results showed more co-localization of macrophages NLRP3 and CD68 in RA synovium (Fig. **3** c-d). Altogether, these results suggest that HSD increases macrophage infiltration in the joint tissue of CIA mice and promotes the development of an inflammatory macrophage phenotype, pyroptosis of macrophages may also be a key factor in promoting the progression of RA disease, a high-salt diet may have adverse effects on RA patients.

### NaCl enhances NLRP3 inflammasome activation and induces inflammatory pyroptosis in macrophages

Research has demonstrated that macrophage pyroptosis, a type of pro-inflammatory programmed cell death, plays a pivotal role in the onset and progression of RA 22. Pyroptosis involves key components such as the NLRP3 inflammasome, GSDMD, IL-1β, IL-18, and other proteins. Overactivation of the NLRP3 inflammasome and pyroptosis leads to the release of inflammatory mediators, ultimately contributing to inflammation, tissue damage, and the exacerbation of RA 23.

**Figure 4:**
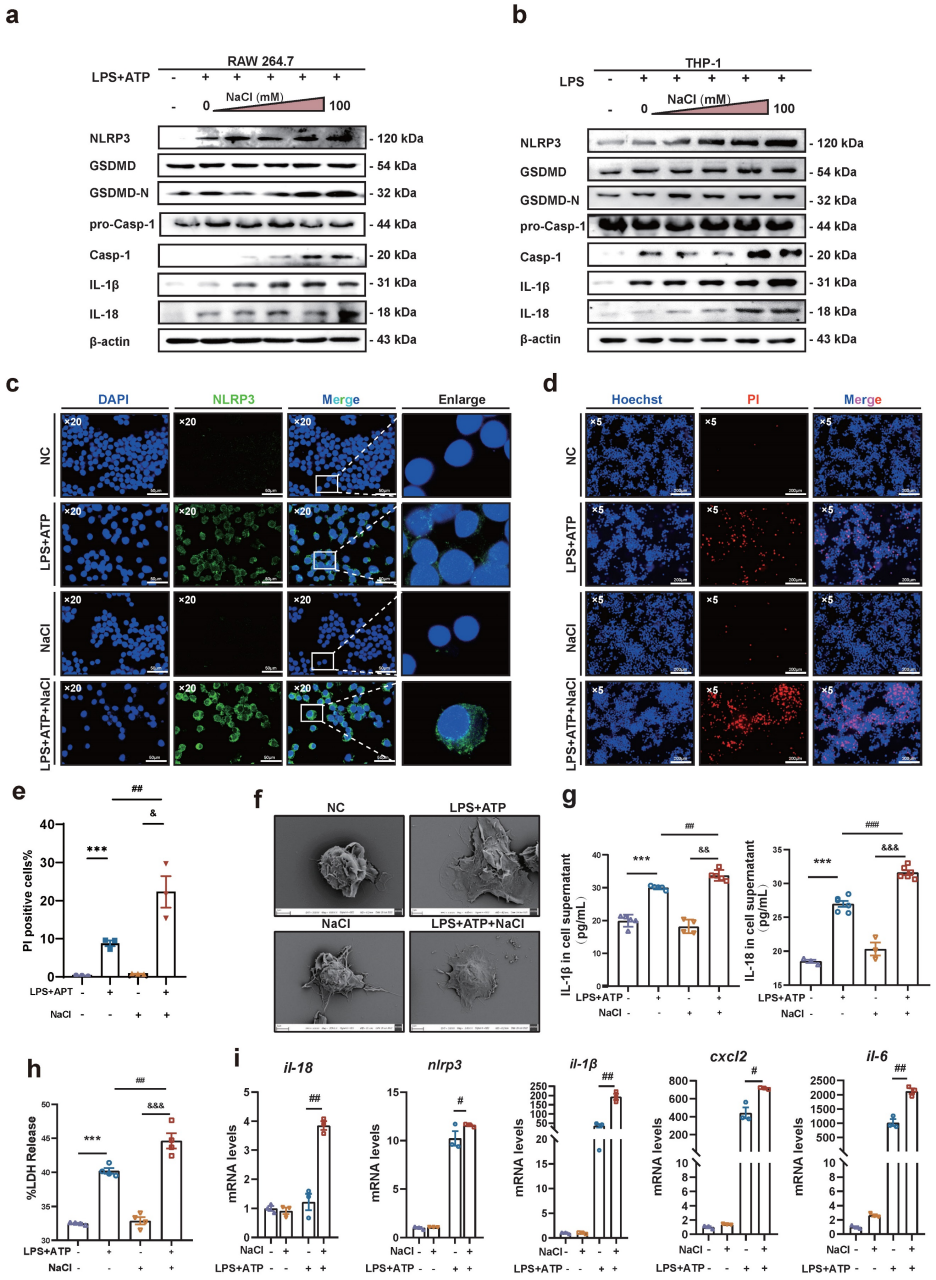
** NaCl activates the expression of NLRP3 inflammasome and the process of pyroptosis in macrophages. (a-b)** Representative western blot images of the effect of sodium chloride on the expression of pyroptosis related proteins NLRP3, GSDMD, caspase1/pro-caspase1, IL-18 and IL-1β in macrophages derived from RAW264.7 (a) and THP-1 cell (b). **(c)** The representative was displayed in confoca immunofluorescence images of expression of NLRP3 protein in control, NaCl, LPS and LPS + NaCl group. (DAPI: blue, NLRP3: green, Scale Bar: 50μm and 10μm). **(d)** Representative images of Hoechst 33342 and PI double fluorescent staining (Hoechst 33342: blue. PI: red, Scale Bar: 200 μm). **(e)** PI uptake statistics of each group (n=3, data are expressed as the mean ± SD). **(f)** The cell morphology of each group was observed by scanning electron microscope (Scale Bar: NC and LPS group: 2μm, NaCl and LPS + NaCl group: 3μm).** (g)** The levels of IL-1β and IL-18 in cell supernatant were detected by ELISA (n=6). **(h)** LDH release levels in RAW264.7 cells after pyroptosis induction and NaCl treatment. Data present as mean ± SD of three independent experiments. ***p<0.001, *##p*<0.01, *&&&p*<0.001 n=5.** (i)** Relative mRNA levels of *Il-18, Nlpr3, Il-1β*,* cxcl6* and *il-6* were detected by RT-qPCR. Data are expressed as the mean ± SD. *#p*<0.05, *##p*<0.01, n=3.

*In vivo* experiments, HSD significantly increased the expression levels of the inflammatory factors IL-1β and IL-18 in the serum of CIA mice, as well as the expression of NLRP3 in macrophages. Therefore, it is conceivable that HSD may exacerbate joint inflammation in CIA by promoting macrophage pyroptosis. To delve deeper into whether NaCl, a component of HSD, is involved in regulating macrophage pyroptosis, RAW264.7 cells (Fig. **4**a) and THP-1 (Fig. **4**b) induced macrophages were pre-treated with varying concentrations of NaCl, followed by induction of pyroptosis with LPS or combine with ATP. Western blot results revealed that NaCl promoted the expression of NLRP3, IL-18, IL-1β, as well as the cleavage of caspase-1 and GSDMD in a concentration-dependent manner, with the most potent effect observed at 50 mM (sFig.**2**). Cell immunofluorescence staining demonstrated that, regardless of NaCl pre-treatment, LPS and ATP stimulated cells exhibited significantly increased NLRP3 inflammasome expression compared to the control group, while NaCl-pretreated cells exhibited even higher NLRP3 inflammasome expression (Fig. **4**c). No significant difference in NLRP3 inflammasome expression was observed between NaCl-pretreated cells and normal cells.

Pyroptosis is characterized by cell swelling and loss of cell membrane integrity. Membrane integrity was assessed using Hoechst/PI staining and LDH release assay. The results indicated that compared to the normal group, the number of PI-positive cells significantly increased in the LPS and ATP stimulated group, indicating successful establishment of the pyroptosis model. Additionally, NaCl pretreatment significantly increased the number of PI-positive cells (Fig. **4**d-e). Furthermore, we observed the morphological changes of cells in each group using scanning electron microscopy (SEM). SEM results showed that RAW264.7 cells treated with LPS exhibited increased adhesion, visible pores on the cell membrane surface, and cells pretreated with NaCl displayed a more typical pyroptotic shape of 'fried egg' with a flatter form (Fig. **4**f). Subsequently, we assessed the levels of IL-1β and IL-18 in cell cultures. As anticipated, NaCl-treated cell cultures exhibited significantly increased production levels of IL-1β and IL-18 (Fig. **4**g). Moreover, LDH release in cell supernatants from all groups demonstrated significantly increased LDH release in cells pretreated with NaCl (Fig. **4**h). These findings suggest that NaCl pretreatment promotes cell membrane rupture and exacerbates pyroptosis. Finally, RT-qPCR results revealed that NaCl pretreatment significantly upregulated the mRNA expression levels of pyroptosis-related genes *Il-1β*, *Il-18*, *Nlrp3*, as well as inflammation-related factors and chemokines *Il-6* and *Cxcl2* in RAW264.7 cells (Fig. **4**i). Cells pretreated with NaCl but not stimulated by LPS showed no significant difference compared to the normal control cells.

### Sodium chloride activation of macrophages involves SGK1, the MAPK/NF-κB signaling pathway and sodium transporter

**Figure 5:**
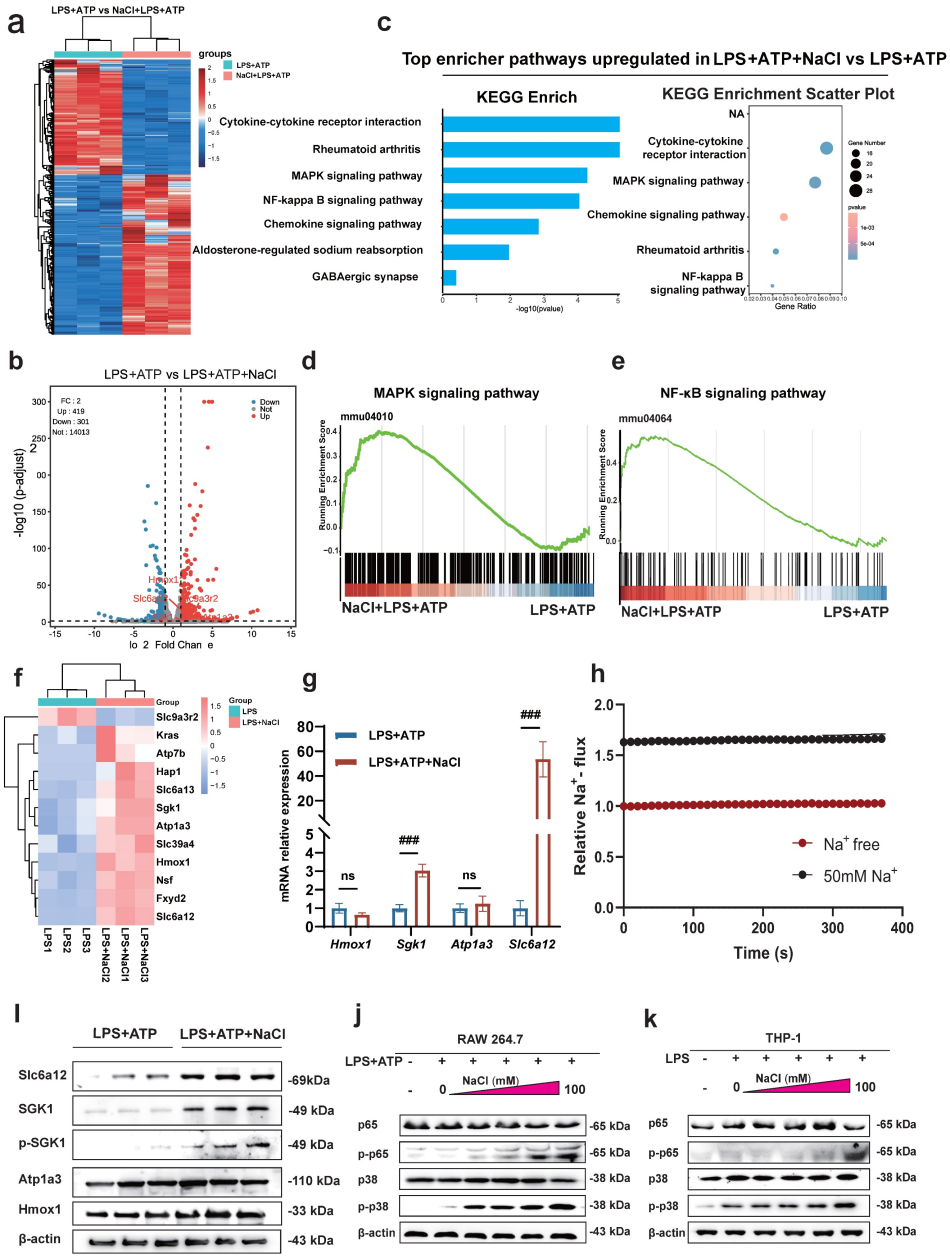
** Sodium chloride activation of macrophages may involve SGK1, the MAPK/NF-κB signaling pathway and sodium transporter. (a)** Heatmap of differential gene changes in Raw264.7 macrophages under LPS and LPS + NaCl treatment detected by RNA-seq. **(b)** Volcano map of differentially expressed genes between pretreated with NaCl (50mM) and LPS-stimulated and LPS-stimulated alone group. Red dots represent up-regulated genes, blue dots represent down-regulated genes, and the genes labeled in the figure are genes related to sodium ion reabsorption. **(c)** KEGG pathway analysis of differentially expressed genes between LPS and LPS + NaCl groups. **(d-e)** Gene set enrichment analysis (GSEA) showing the cells pretreated with NaCl (50mM) enriched the MAPK **(d)** and NF-κB **(e)** pathway compared with the LPS+ATP group. **(f)** The heat map of significantly changed genes associated with sodium reabsorption. **(g)** RT-qPCR was used to detect the gene changes closely related to sodium ion. Data are expressed as the mean ± SD. *###p*<0.001, n=3. **(h)** The SoNa 520 AM system was used to detect macrophage Na^+^ influx under treatment with 50 mM or free conditions. **(i)** Representative western blot images of the effect of sodium chloride on the expression of Slc6a12, SGK1, p-SGK1, Atp1a3 and Hmox1in RAW264.7 cell. **(j-k)** Western blot was used to examine the effect of different concentration of NaCl on NF-κB p65 and MAPK p38 phosphorylation in Raw264.7 and THP-1-derived macrophages.

To uncover the specific mechanism by which Na^+^ affects macrophages, we conducted RNA-seq to compare the transcriptomes of LPS and ATP treated RAW264.7 cells with or without NaCl pretreatment. The results revealed that compared to the LPS and ATP treated group, there were 419 upregulated genes and 301 downregulated genes in cells pretreated with NaCl (Fold change > 1, FDR < 0.05) (Fig. **5**a-b). To explore the functional relationships between differentially expressed genes, we performed Kyoto Encyclopedia of Genes and Genomes (KEGG) pathway enrichment analysis, which highlighted the most significant changes in LPS and ATP stimulated RAW264.7 cells after NaCl pretreatment. Notably, processes related to inflammation, such as 'Cytokine-cytokine receptor interaction' and the 'Chemokine signaling pathway', exhibited significant alterations. Additionally, NaCl pretreatment significantly affected genes associated with RA, aligning with our previous *in vivo* experimental findings, thus confirming our initial hypothesis. Importantly, the addition of NaCl enriched and upregulated the MAPK signaling pathway and the NF-κB signaling pathway (Fig. **5**c-e). In order to further explore the direct factors affecting the response of macrophages by Na^+^ in NaCl, we analyzed genes related to sodium reabsorption among the differentially expressed genes, as depicted in heat maps and volcano plots (Fig. **5**f). Simultaneously, in order to identify the key influencing genes of Na^+^, RT-qPCR was used to verify the results, and it was found that *Sgk1* (*P*=0.0068) and *Slc6a12* (*P*=0.0093) genes were most significantly elevated after NaCl pretreatment compared with cells stimulated by LPS and ATP alone (Fig. **5g**). Western blot experiments showed that the protein levels of SGK1 and Slc6a12 in THP-1 cells significantly increased after the addition of 50mM NaCl, consistent with the RT-qPCR results (Fig. **5**i). SGK1 and Slc6a12 are closely linked to sodium ions, SGK1, a serum and glucocorticoid-induced kinase, not only regulates sodium ion channels, controlling the influx and efflux of sodium ions, but also acts as a central player in various signaling pathways and cellular phosphorylation, strongly associated with inflammation 24. On the other hand, Slc6a12, also known as BGT-1, is a transporter responsible for clearing the inhibitory neurotransmitter gamma-aminobutyric acid (GABA) from the synaptic gap. It utilizes the concentration gradient of sodium ions as an energy source to transport both GABA and sodium ions into the cell simultaneously, effectively serving as a sodium ion transporter 25. Thus, further investigation is warranted to elucidate the precise roles of these two significantly altered genes in the context of Na^+^ effects on macrophages. SoNa 520am was used to detect transient changes in intracellular Na^+^ levels with or without addition of 50 mM sodium chloride, and found that addition of 50 nm did significantly increase an intracellular Na^+^ flux (Fig. **5**h). Remarkably, the MAPK/NF-κB signaling cascade is closely linked to the activation of the NLRP3 inflammasome 26,27.

To validate these results, we used mouse RAW264.7 cells and human THP-1 cells to confirm that NaCl significantly increased the expression of p-P38 and p-P65 in cells stimulated by LPS and ATP in a concentration-dependent manner (Fig. **5**j-k and sFig **3**). In summary, these results highlight the intricate relationship between Na^+^ and Na^+^-sensitive genes, such as *Sgk1* and *Slc6a12*, within the context of the p38 MAPK/NF-κB signaling pathway.

### Knock down of SGK1 reverses sodium-mediated stimulation of macrophage pyroptosis through the p38 MAPK/NF-κB signaling pathway

**Figure 6:**
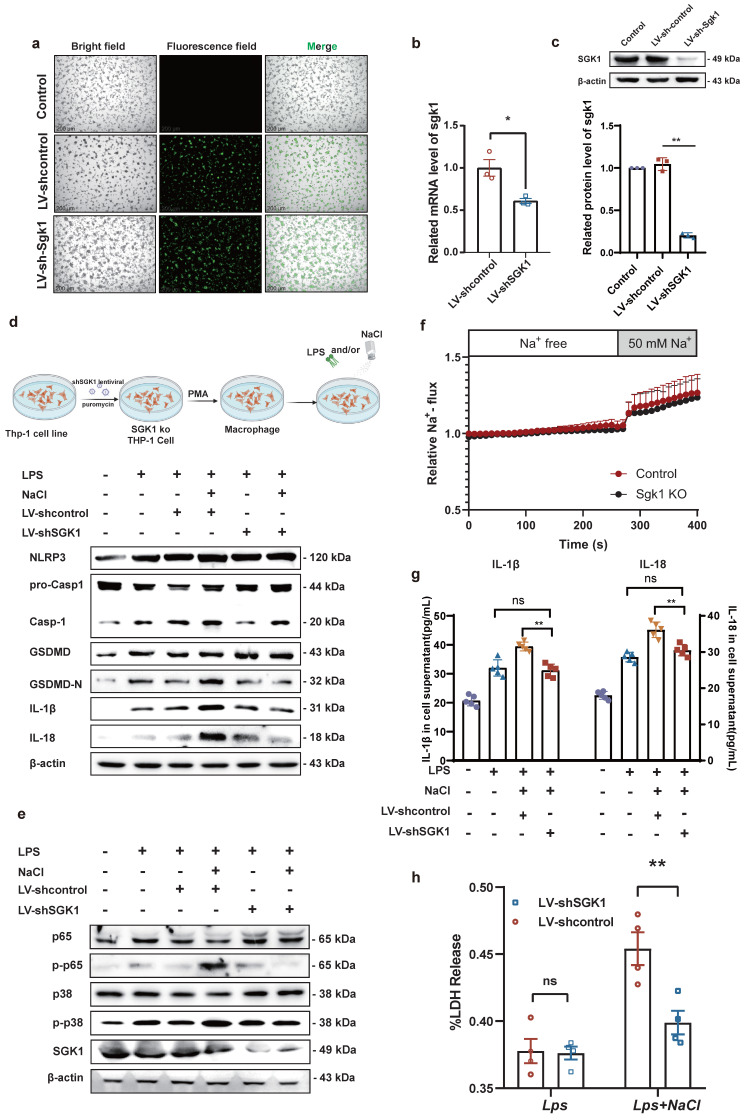
** SGK1 knockout inhibits the activation of macrophage pyroptosis by NaCl through the MAPK/NF-κB pathway.** THP-1 cells were infected with lentivirus containing an SGK1 (LV-shSGK1) expression plasmid or an empty vector (LV-control) that confers resistance to puromycin.** (a)**The image of LV-control and LV-SGK1 group transfected for 72h and screened with puromycin (The lentivirus carries the EGFP label with green fluorescent) (Scale Bar: 200 μm). **(b-c)** RT-qPCR and Western blot analysis were performed on THP-1 cells with stable knock down of SGK1 expression (n=3). Data are expressed as the mean ± SD. *p<0.05, **p<0.01. **(d-e)** Representative western blot images of the effect of sodium chloride on the expression of pyroptosis related protein** (d)**, NF-κB p65 and MAPK p38 **(e)**. **(f)** The SoNa 520 AM system was used to detect macrophage Na^+^ influx in SGK1 knock down macrophages. **(g)** ELISA was used to detect SGK1 knock down on the effect of IL-1β and IL-18 in cell supernatant (n=6). **(h)** Effect of NaCl treatment on LDH release levels in culture supernatants of SGK1-deficient macrophages. Data are expressed as the mean ± SD. **p<0.01, n=4.

Previous studies have shown that MAPK and NF-κB play an important role in the inflammatory response and the activation of the NLRP3 inflammasome 28. Base on this, our prior evidence has demonstrated that NaCl promotes pyroptosis while simultaneously activating SGK1 and the p38 MAPK/NF-κB signaling cascades. According this, we hypothesized that NaCl could enhance macrophage pyroptosis by activating the p38 MAPK/NF-κB signaling pathway following SGK1 activation. To investigate this hypothesis, we employed SGK1 shRNA-expressing lentivirus (Lv-shSGK1) to silence SGK1 expression in THP-1 cells. We introduced SGK1 shRNA-expressing lentivirus and negative control virus (LV-Control), both labeled with EGFP, into THP-1 cells (Fig. **6**a), followed by puromycin-based screening to establish stable cell lines. Subsequently, Western blot and RT-qPCR analyses confirmed significant reductions in SGK1 transcriptional and protein levels, indicative of successful stable cell line construction (Fig. **6**b-c).

Notably, silencing SGK1 markedly reduced the expression of pyroptosis-related proteins under the pretreaed by NaCl and LPS. The expression of NLRP3, IL-1β, IL-18, GSDMD, and cleaved caspase-1 assessed the levels of pyroptosis were detected. Western blot analysis revealed that in cell lines with negative viral transfections, NaCl significantly increased the expression levels of these pyroptosis-related proteins when combined with LPS stimulation (Fig. **6**d and sFig **4**). Importantly, NaCl did not elevate the expression of p-P38 MAPK or key proteins within the NF-κB signaling cascade, namely p-P38 and p-P65, in SGK1 shRNA-expressing lentivirus stable cell lines (Fig. **6**e and sFig **4**). The absence of SGK1 did not seem to affect the inflow of Na^+^, which was confirmed by the SoNa 520 AM results (Fig. **6**f). Furthermore, by collecting cell culture supernatant, ELISA results further indicated that the loss of SGK1 could down-regulate the levels of IL-1β and IL-18 under LPS combined with NaCl pretreatment (Fig. **6**g). LDH release results also confirm that, in negative lentivirus-transfected cell lines, NaCl pretreatment led to a significant increase in LDH release compared to the group stimulated solely by LPS. However, the silencing of SGK1 mitigated the NaCl-induced rise in LDH release (Fig. **6**h). Consequently, the inhibition of SGK1 effectively reversed the influence of NaCl on macrophage pyroptosis, and this reversal was achieved through the mediation of the p38 MAPK/NF-κB signaling pathway.

### Overexpression of SGK1 rescues the promotion of pyroptosis which is caused by sodium transported by Slc6a12 in SGK1 silenced cells

**Figure 7:**
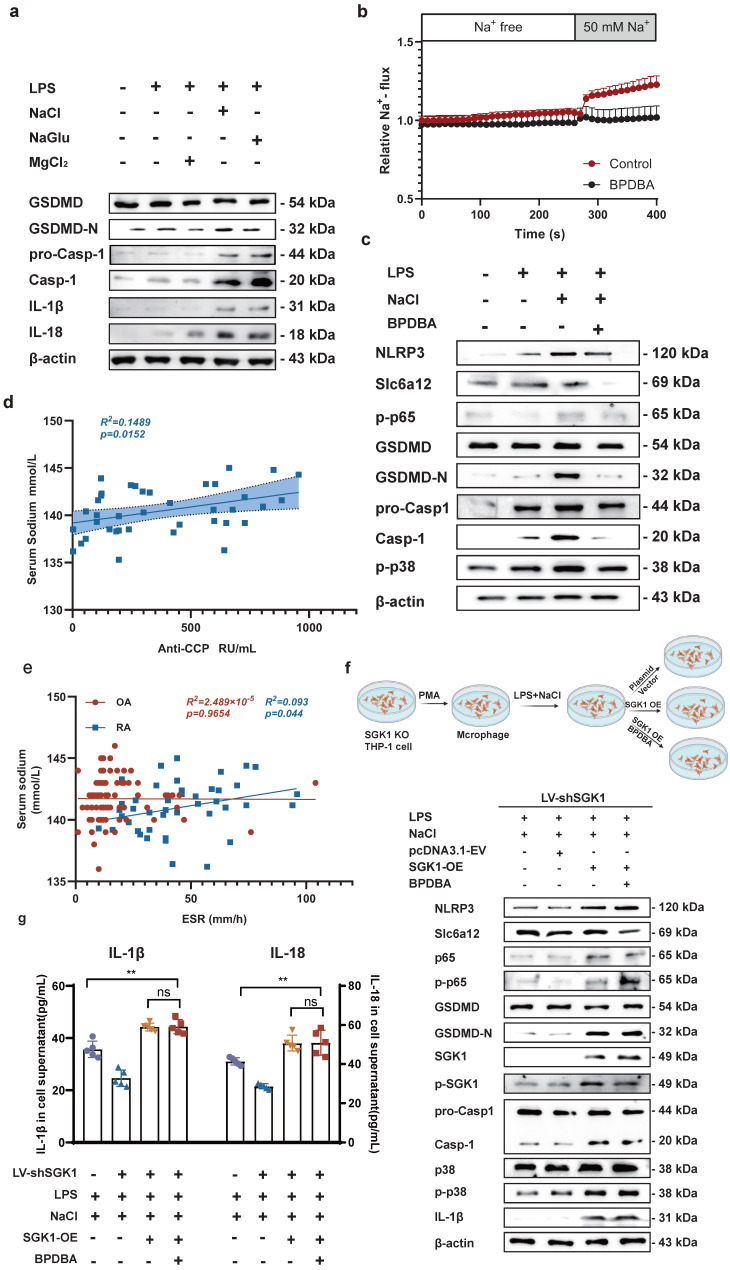
** Na^+^ enters the cell through Slc6a12 transporter to activate SGK1 and activate MAPK signaling pathway to promote macrophage pyroptosis. (a)** Western blot analysis of Caspase-1, IL-18 and IL-1β protein expression of RAW264.7 cells after adding magnesium chloride, sodium chloride and sodium gluconate. **(b)** The SoNa 520 AM system was used to assay the effect of the Slc6a12 inhibitor BPDBA on Na^+^ influx into THP-1-derived macrophages. **(c)** The effects of Slc6a12 inhibitor (BPDBA) on Sodium-activated p38 MAPK/NF-κB and were detected by western blot. **(d)** Scatter plot and regression line between serum sodium content and anti-cyclic citrulline antibody value in RA patients. **(e)** Correlation analysis between serum Sodium level and erythrocyte sedimentation rate in patients with RA and OA. **(f)** In SGK1-deficient macrophages pretreated by LPS + NaCl followed by SGK1 overexpression or BPDBA treatment, western blot was used to detect the effects of pyroptosis, NF-κB p65 and MAPK p38 related protein expression. **(g)** ELISA was used to detect IL-1β and IL-18 levels in culture supernatants of SGK1-deficient macrophages under SGK1 rescue or BPDBA treatment. Data are expressed as the mean ± SD, n=5.

To identify the key ions in NaCl responsible for these effects, we pretreated RAW264.7 cells with different isotonic compounds (NaCl, NaGlu, MgCl_2_), followed by LPS-induced pyroptosis. Western blot results demonstrated that isotonic sodium chloride and sodium gluconate pretreatment significantly increased the expression of IL-18, IL-1β, caspase-1, and GSDMD. However, this effect was not observed in cells treated with magnesium chloride (Fig. **7**a and sFig 5). These findings indicated that Na^+^ was the key ion responsible for promoting macrophage pyroptosis. Therefore, we speculated that inhibiting the expression of Slc6a12 could inhibit the transport of Na^+^ and thus inhibit the pyroptosis of macrophages induced by Na^+^. In order to further investigate the effect of Na^+^ transporter Slc6a12 on macrophage pyroptosis, we used BPDBA (Slc6a12 selective inhibitor) to pretreat THP-1-induced macrophages, our result dedicated that 20 μM BPDBA had no effect on the proliferation of THP-1 macrophage (sFig**6**). The SoNa 520 AM system showed that BPDBA significantly inhibited Na^+^ inflow (Fig. **7**b). Western blot results demonstrated that NaCl enhanced the expression of pyroptosis-related proteins, as well as p-P38 and p-P65, while the addition of BPDBA inhibited this promotional effect (Fig. **7**c and sFig **7**). In additional, the relationship between Na^+^ levels and RA was evaluated, interestingly, the results indicated a positive correlation between serum sodium concentration in RA patients and anti-cyclic citrulline antibodies (anti-CCP) (*P*=0.0152, *r*=0.3859) (Fig. **7**d). Serum sodium concentration in RA patients was positively correlated with erythrocyte sedimentation rate (ESR) (*P*=0.044, *R^2^*=0.093), whereas serum sodium concentration in OA patients showed no correlation with ESR (Fig. **7**e). Additionally, to further elucidate the specific role of SGK1, we introduced an SGK1-overexpressing plasmid and an empty plasmid into SGK1 knock down THP-1 cell lines.

Knock down SGK1 in THP-1-induced macrophages were subjected to overexpression of SGK1 for a period of 24 hours, followed by treatment with NaCl and/or LPS. Western blot analysis revealed that SGK1 overexpression restored the promoting effect of Na^+^ on macrophage pyroptosis, concurrently elevating the expression of p-P38 and p-P65 (Fig. **7**f). At the same time, Slc6a12 inhibitor BPDBA was added after SGK1 overexpression, and the results showed that BPDBA could not reverse the expression of p-P38, p-P65, NLRP3, IL-1β, IL-18, GSDMD-N, cleaved caspase-1 (Fig. **7**f and sFig **8**). Analysis of macrophage culture supernatants from each group by ELISA also confirmed that SGK1 was downstream of Slc6a12, realizing that the levels of IL-1β and IL-18 were significantly elevated after overexpression of SGK1 by the co-treatment of LPS and NaCl, and that BPDBA was unable to reverse this part of the effect, which was in accordance with the WB results (Fig. **7**g). This suggests that Slc6a12 serves as the upstream channel regulating SGK1 and macrophage pyroptosis. In conclusion, these findings suggest that NaCl, particularly Na^+^ entering the cells through the Na^+^ transporter Slc6a12 then activates SGK1, promotes the expression of the NLRP3 inflammasome, exacerbates membrane rupture and leakage, and enhances the production of related inflammatory factors, thereby causing inflammatory damage.

## Discussion

Sodium chloride, ubiquitously used as a seasoning, not only enhances the palatability of food but is also crucial for maintaining electrolyte balance within the body. Nonetheless, dietary patterns marked by excessive salt consumption significantly exceed the guidelines recommended by the World Health Organization. Chronic intake of high-salt diets has been associated with a spectrum of health issues, including vascular diseases, obesity, nephropathy 29, various cancers, and neurodegenerative conditions such as Alzheimer's disease 30,31. The influence of high-salt diets on the pathogenesis of autoimmune disorders has garnered heightened scrutiny. Studies reveal that an environment rich in sodium chloride potentiates the differentiation of pro-inflammatory Th17 cells and elevates the expression of cytokines including granulocyte-macrophage colony-stimulating factor (GM-CSF) and TNF-α. These molecular perturbations are thought to escalate disease severity, exemplified by conditions such as experimental autoimmune encephalomyelitis (EAE), a model for multiple sclerosis 32.

Clinical observations bolster these insights, with epidemiological evidence establishing a positive correlation between dietary sodium intake and the rate of progression in multiple sclerosis (MS) 33. In the realm of inflammatory bowel disease (IBD), HSD have been shown to precipitate the synthesis of interleukin-23 (IL-23), concomitantly enriching the population of IL-17-producing type 3 innate lymphoid cells (ILC3s), thus aggravating intestinal inflammation 34,35. Furthermore, investigations in murine models indicate that elevated salt consumption can perturb gut microbiota composition, diminishing the prevalence of lactobacillus and the production of butyrate—a short-chain fatty acid with anti-inflammatory properties. This shift is accompanied by upregulated expression of pro-inflammatory genes such as ATF2, which intensifies colitis symptoms in these animals 36. Recent findings suggest that HSD may influence the onset and progression of RA. Physiological assessments reveal that human bones are rich in sodium ions, with magnetic resonance imaging (MRI) quantification indicating concentrations ranging from 115-150 mmol/L in non-cartilaginous regions to 200-210 mmol/L within cartilage areas, underscoring a fundamental link between bone physiology and sodium levels 37. Notably, a high intake of sodium is correlated with the pathophysiology of RA, manifesting in heightened sodium excretion among patients 38. Moreover, the detrimental role of sodium in RA is echoed in animal studies; for instance, in models of CIA and K/BxN serum transfer arthritis, mice subjected to a low-salt diet (LSD) exhibited attenuated inflammatory infiltration within the joints and diminished cartilage erosion compared to their HSD-fed counterparts 39. These observations align with our laboratory experimental results. However, the work of Sehnert and colleagues, while corroborating these findings, did not extend to a mechanistic exploration of how elevated sodium intake exacerbates disease activity in murine models.

In our investigation, we uncovered a significant positive correlation between serum sodium ion concentration and critical RA biomarkers, such as anti-CCP antibodies and ESR, in patients with RA. This association did not present in individuals with OA. Our *in vivo* and *in vitro* assessments revealed that an HSD intensifies joint inflammation and cartilage damage in a CIA mouse model. Additionally, NaCl was observed to accelerate the advancement of pyroptosis in macrophages. Macrophages exposed to elevated NaCl levels demonstrated an upsurge in the secretion of pro-inflammatory cytokines, amplified expression of immunostimulatory molecules, and increased proliferation of antigen-independent T cells. Conversely, a high-salt milieu inhibits the alternative activation of bone marrow-derived macrophages induced by interleukin-4 (IL-4) and interleukin-13 (IL-13), thereby diminishing their capacity to curb the expansion of effector T cells 40. Intriguingly, the work of He et al 41. revealed that a high-salt diet may impede tumorigenesis in mice, possibly by promoting the differentiation of myeloid cells within the tumor microenvironment into pro-inflammatory phenotypes. The study acknowledges that, while HSD may play a beneficial role in the context of cancer, such dietary habits are disadvantageous in RA, where HSD-induced macrophage activation could potentially accelerate osseous degradation.

Macrophages, as key effectors of the innate immune system, are central to the pathogenesis of RA—a disease whose etiology, despite being multifaceted and enigmatic, notably involves the expansion of macrophage subsets within the synovial membrane, signaling the nascent phase of RA and characterizing the inflammatory lesions 10, 42. These macrophages predominately adopt a pro-inflammatory phenotype, instigating a cascade of inflammation through the production of pro-inflammatory mediators, attracting and activating further immune cells, and contributing to the destruction of cartilage and bone. Our team's previous investigations established macrophage pyroptosis as a critical driver in RA pathogenesis 43,44. In our current study, we delved into the mechanistic underpinnings of HSD-induced macrophage pyroptosis. Employing functional enrichment analyses, we delineated that NaCl prompts the activation of the MAPK/NF-κB signaling pathway in RAW264.7 cells, with prior research indicating the regulatory role of the p38 MAP kinase in response to hyperosmolarity, leading to the activation of protective cellular mechanisms and inflammation-associated signaling cascades. We have discovered that the p38 MAPK pathway can trigger the NLRP3 inflammasome, primarily by facilitating nuclear translocation of the p65 subunit of NF-κB—pivotal for transcriptional activation of pro-inflammatory genes 45. Further, our results provide novel insights into the way NaCl can instigate the NLRP3 inflammasome through the p38 MAPK/NF-κB signaling axis. This is evidenced by the observed concentration-dependent elevation of phosphorylated p38 and p65 proteins in macrophage cell lines, both human and murine, thus endorsing the proposed inflammatory mechanism and broadening our comprehension of salt's exacerbatory effects in RA.

**Figure 8:**
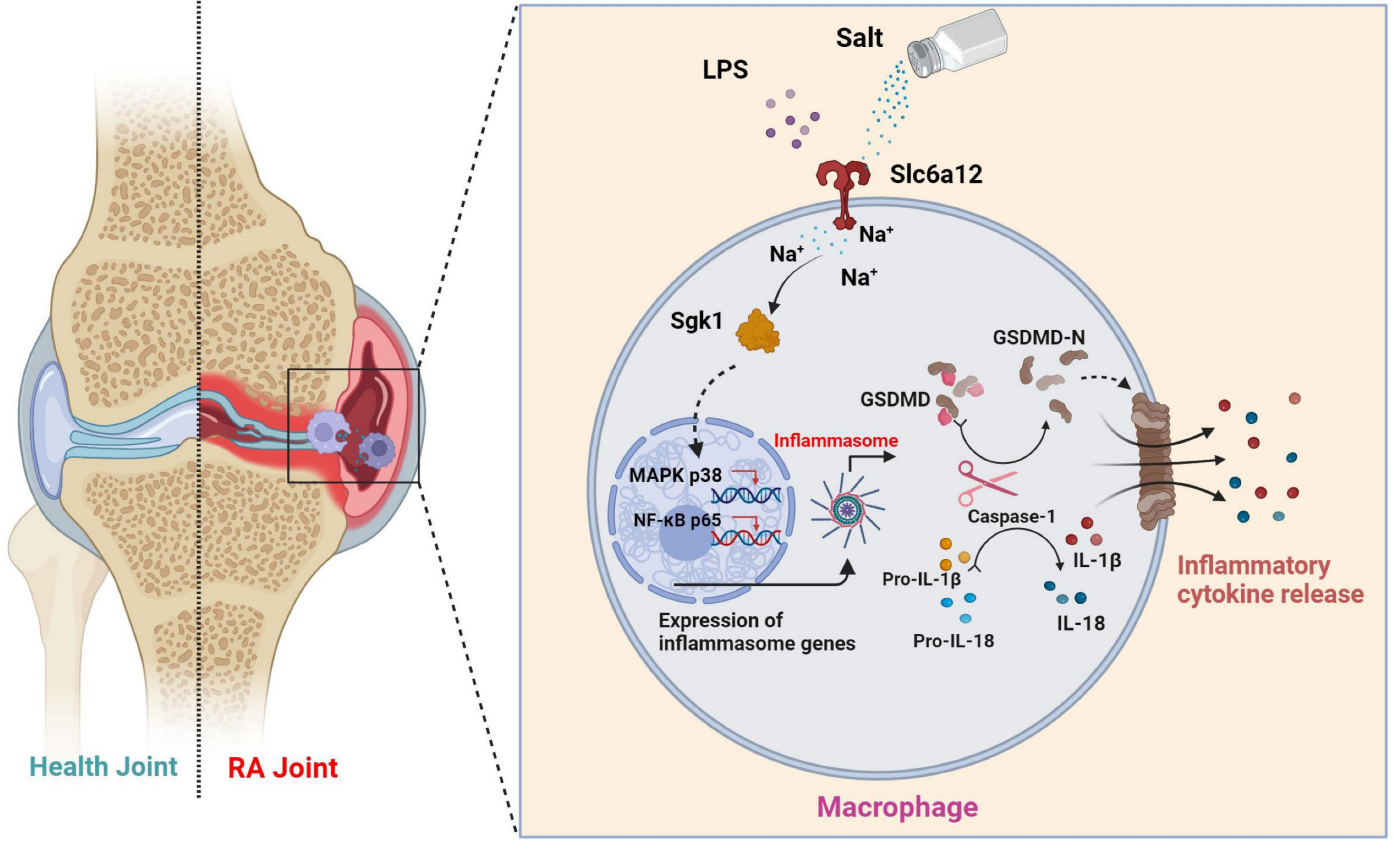
** Schematic illustration of high salt diet promoting RA progression:** high concentration of sodium ions enters into macrophages in synovial tissues through the sodium ion transporter Slc6a12, which activates SGK1 by promoting phosphorylation of SGK1, followed by the production of large amounts of inflammatory cytokines IL-1β and IL-18 by promoting classical pyroptosis pathway through the MAPK p38 and NF-κB p65 signaling pathways, which aggravates the synovial inflammation.

To elucidate the direct effects of sodium ions on macrophage function, our investigation targeted genes whose expression was altered in concert with sodium ion fluctuations. Intriguingly, Terada et al. identified SGK1 as a sensor of extracellular sodium levels that promotes differentiation of Th17 cells under high-sodium conditions. Activation of SGK1 correlates with heightened levels of pro-inflammatory cytokines, including IL-1β and IL-6, in mesangial cells 46. Additionally, macrophage-specific deletion of the mineralocorticoid receptor attenuates NF-κB activation, an effect shown to be SGK1-dependent 47. Nonetheless, the involvement of SGK1 in macrophage-mediated inflammation warrants further examination. We proposed that the activation of SGK1 by sodium ions might trigger macrophage pyroptosis—an avenue ripe for exploration. The entry mechanism of sodium ions into macrophages, however, remains an unresolved dimension of their downstream effects. We considered the potential role of Betaine/gamma-aminobutyric acid (GABA) transporter 1 (BGT-1 or Slc6a12), a recognized GABA and osmolyte betaine transporter that utilizes sodium ion gradients across cellular membranes 48. Slc6a12 is implicated in the cellular uptake of two sodium ions alongside one chloride ion upon reaching a specific extracellular concentration gradient—indicative of its function as a sodium ion transporter. Our observations indicated that pharmacological inhibition of Slc6a12 curtailed the influence of NaCl on p38 MAPK and p65 nuclear translocation, as well as the expression of proteins associated with pyroptosis—in normal, but not SGK1-silenced cells with restored SGK1 expression. These findings support a model wherein sodium ions, upon their entry into macrophages via Slc6a12, activate SGK1, which in turn precipitates macrophage pyroptosis through the p38 MAPK/NF-κB signaling pathway, thereby contributing to the pathophysiology of RA (Fig 8). Thus, targeting the sodium ion channel signaling cascade emerges as a promising strategy for mitigating macrophage pyroptosis and represents an insightful starting point for the synthesis of new RA therapeutics. Moreover, our research reinforces the adverse role of high-sodium diets in RA progression and underscores the health imperative of reduced salt intake—particularly for individuals grappling with autoimmune conditions like RA.

Our investigation offers a comprehensive assessment of how an HSD influences the pathogenesis of RA. Focused on the perturbations caused by NaCl, a principal constituent of HSD, we have dissected its roles in macrophage pyroptosis, delineating the precise mechanisms by which NaCl modulates this form of programmed cell death. Despite these insights, our research is not without limitations. To fully delineate the implications of HSD on macrophage function within the RA milieu, additional experiments are imperative. A promising direction for future enquiry involves subjecting macrophage SGK1-conditional knockout mice within a CIA framework to diets of varying sodium content. Such approaches will allow us to probe the nuanced roles of SGK1 in shaping macrophage responses under high-salt conditions and further clarify the molecular interplay driving the progression of RA in the context of dietary sodium.

## Supplementary Material

Supplementary figures.

## Figures and Tables

**Table 1:** The specific sequences and primers used in this investigation.

Gene	F/R	Sequence
*Mice-F4/80*	F	5'- CTGCACCTGTAAACGAGGCTT-3'
	R	5'-GCAGACTGAGTTAGGACCACAA-3'
*Mice-Il-6*	F	5'- TTGCCTTCTTGGGACTGATG-3'
	R	5'- TCATTTCCACGATTTCCCAG-3'
*Mice-Nlrp3*	F	5'- CAAGGCTGCTATCTGGAGGAA-3'
	R	5'- TGCAACGGACACTCGTCATC-3'
*Mice-Il-1β*	F	5'- TGCCACCTTTTGACAGTGATG-3'
	R	5'- TGATGTGCTGCTGCGAGATT-3'
*Mice-Cd80*	F	5'- TTCACCTGGGAAAAACCCCC-3'
	R	5'- ACAACGATGACGACGACTGT-3'
*Mice-Cd86*	F	5'-CAGCACGGACTTGAACAACC-3'
	R	5'- CTCCACGGAAACAGCATCTGA-3'
*Mice-Cd206*	F	5'- TTCAGCTATTGGACGCGAGG-3'
	R	5'- GAATCTGACACCCAGCGGAA-3'
*Mice-Arg-1*	F	5'- CGTAGACCCTGGGGAACACTAT-3'
	R	5'- TCCATCACCTTGCCAATCCC-3'
*Mice-Cxcl2*	F	5'- TCCAAAAGATACTGAACAAAGGC-3'
	R	5'- TTTGGTTCTTCCGTTGAGGG-3'
*Mice-β-Actin*	F	5'- CACTGTCGAGTCGCGTCC-3'
	R	5'- TCATCCATGGCGAACTGGTG-3'
*Human-Hmox1*	F	5'- GGGTGATAGAAGAGGCCAAGA-3'
	R	5'- AGCTCCTGCAACTCCTCAAA-3'
*Human-Sgk1*	F	5'- GCAGAAGAAGTGTTCTATGCAGT-3'
	R	5'- CCGCTCCGACATAATATGCTT-3'
*Human-Atp1a3*	F	5'- AAGATGCAGGTGAACGCTGA-3'
	R	5'- GGGAGGAGTTGTCCACCTTG-3'
*Human-Slc6a12*	F	5'- GGAGAAACCTCGGGGCAT-3'
	R	5'- AGGGCCAAAGCCAAGACAA-3'
*Human-Gapdh*	F	5'- GAGTCAACGGATTTGGTCGT-3'
	R	5'- GACAAGCTTCCCGTTCTCAG-3'
