# Supplementary material for: Sodium chloride promotes macrophage pyroptosis and aggravates rheumatoid arthritis by activating SGK1 through GABA receptors Slc6a12
Source: Int J Biol Sci. 2024 May 11;20(8):2922–42. doi: 10.7150/ijbs.93242 (PMC11186373; doi:10.7150/ijbs.93242)
Supplement: Supplementary file 1 — Supplementary figures. [file ijbsv20p2922s1.pdf]

Supplementary Figures & legends

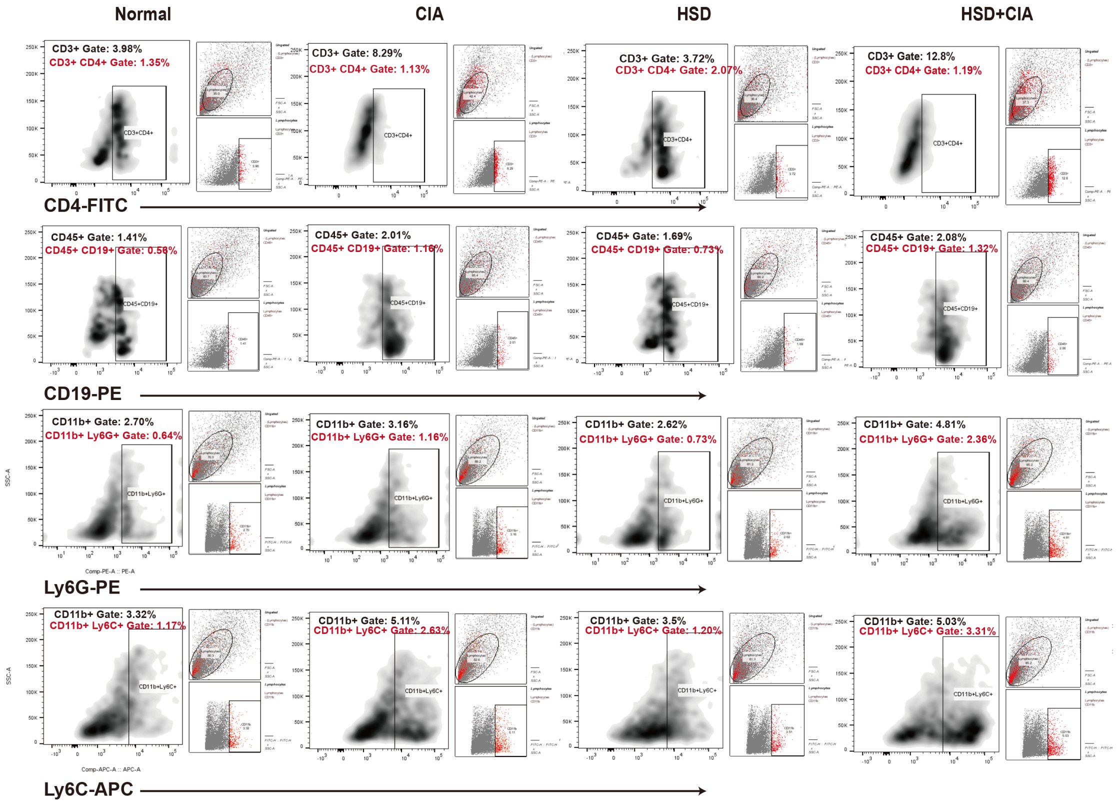

Supplementary Fig1. The gating strategy and represent image for flow cytometry. The proportion of CD3$^+$ T cell, CD19$^+$ B cells, CD11b$^+$Ly6G$^+$ neutrophils, CD11b$^+$Ly6C$^+$ monocytes and macrophages in mice paws assessed using flow cytometry. As illustrated, the combination of HSD and CIA led to a heightened level of immune cell infiltration in the paws compared to the CIA group.

sFig 2

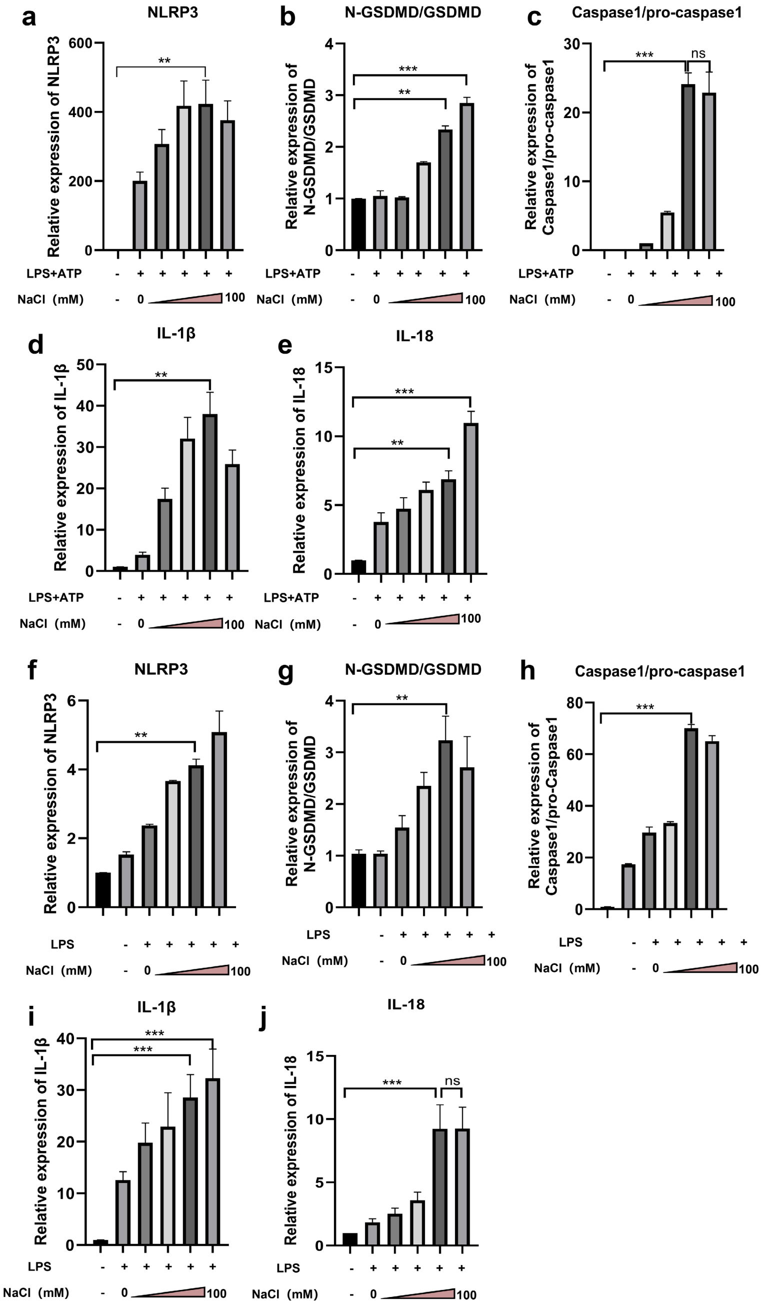

Supplementary Fig2. Quantitative analysis of western blot bands on the impact of sodium chloride on macrophage pyroptosis. (a-e) The impact of sodium chloride on the expression of NLRP3 **(a)**, GSDMD-N **(b)**, Caspsase1 **(c)**, IL-1β **(d)** and IL-18 **(e)** in Raw264.7 macrophages under LPS combined with ATP priming conditions. **(f-j)** The impact of sodium chloride on the expression of NLRP3 **(f)**, GSDMD-N **(g)**, Caspsase1 **(h)**, IL-1β **(i)** and IL-18 **(j)** in THP-1 induced macrophages under the treatment of LPS. Data are expressed as the mean ± SD, n=3. ** p<0.01, *** p<0.001.

sFig 3

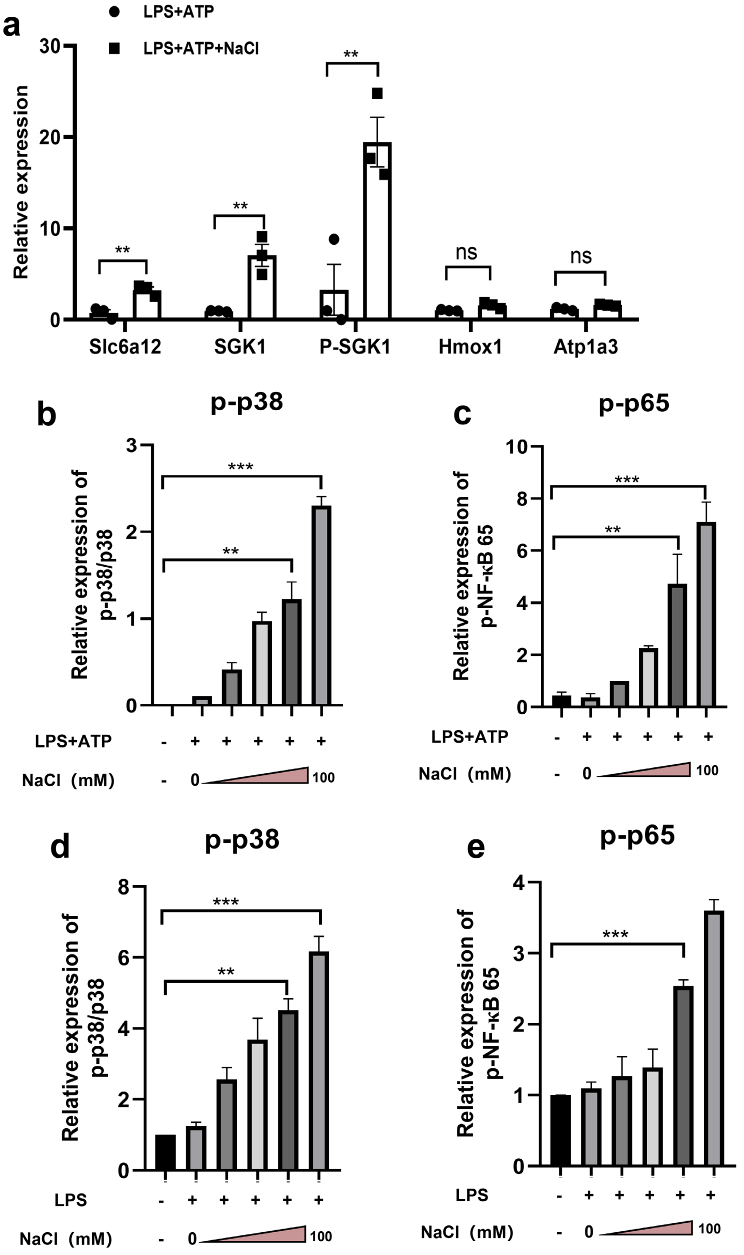

Supplementary Fig3. Quantitative Western blot analysis of Na+ transport proteins and MAPK/NF-kappaB under the treatment of 50 mM NaCl. **(a)** Quantitative Western blot analysis of the effects of NaCl on the expression of Slc6a12, SGK1, p-SGK1, Hmox1 and Atp1a3 proteins based on RNA-seq results. **(b-c)** Quantitative Western blot analysis of the effects of NaCl on the phosphorylation of MAPK p38 **(b)** and NF-κB p65 **(c)** in Raw264.7 cells. **(d-e)** Quantitative Western blot analysis of the effects of NaCl on the phosphorylation of MAPK p38 **(d)** and NF-κB p65 **(e)** in THP-1induced macrophages. Data are expressed as the mean ± SD, n=3. ** p<0.01, *** p<0.001.

sFig 4

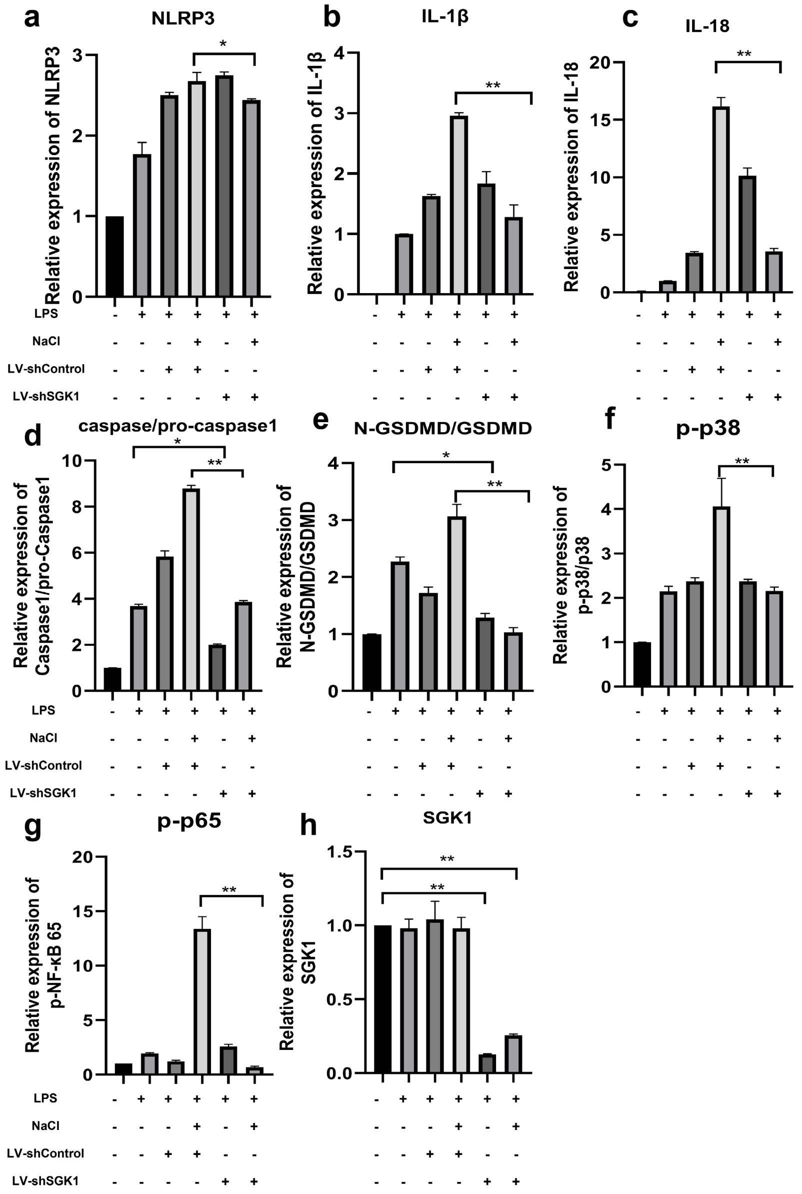

Supplementary Fig4. Quantitative Western blot analysis of the effects of SGK1 knockout on the expression of apoptosis-related proteins in THP-1 derived macrophages. (a-e) Quantitative Western blot analysis of the effects of SGK1 knockout on the expression of proteins NLRP3**(a)**, IL-1β**(b)**, IL-18**(c)**, caspase1**(d)** and N-GSDMD **(e)** under treatment NaCl and LPS. **(f-h)** Quantitative Western blot analysis of the effects of SGK1 knockout on the expression of proteins SGK1 **(h)** and phosphorylation of MAPK p38 **(f)** and NF-κB p65 **(g)** under treatment NaCl and LPS in THP-1induced macrophages. Data are expressed as the mean ± SD, n=3. * p<0.05, ** p<0.01.

sFig 5

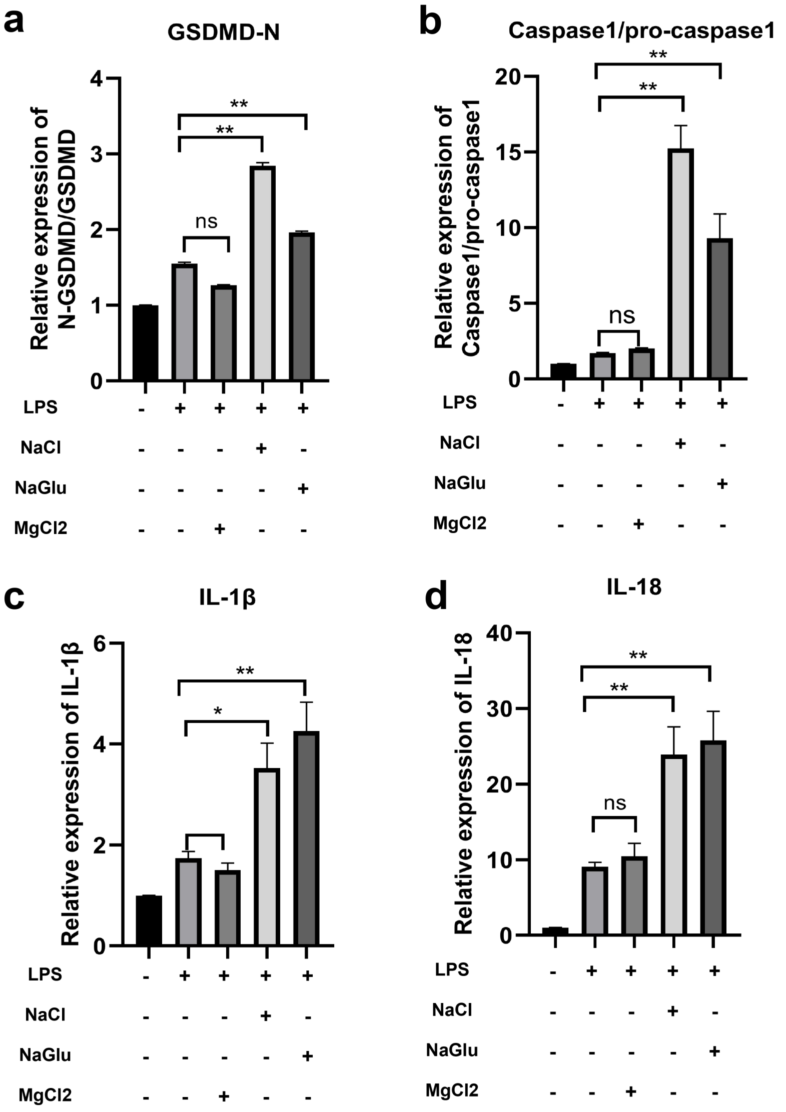

Supplementary Fig5. Quantitative Western blot analysis of the effects of sodium ions, magnesium ions, and chloride ions on pyroptosis of THP-1 derived macrophages. (a-d) Quantitative Western blot analysis of the effects of pre-treatment with different ions on the expression of proteins GSDMD-N**(a)**, caspase1**(b)**, IL-1β**(c)** and IL-18**(d)** in THP-1 macrophages.

sFig 6

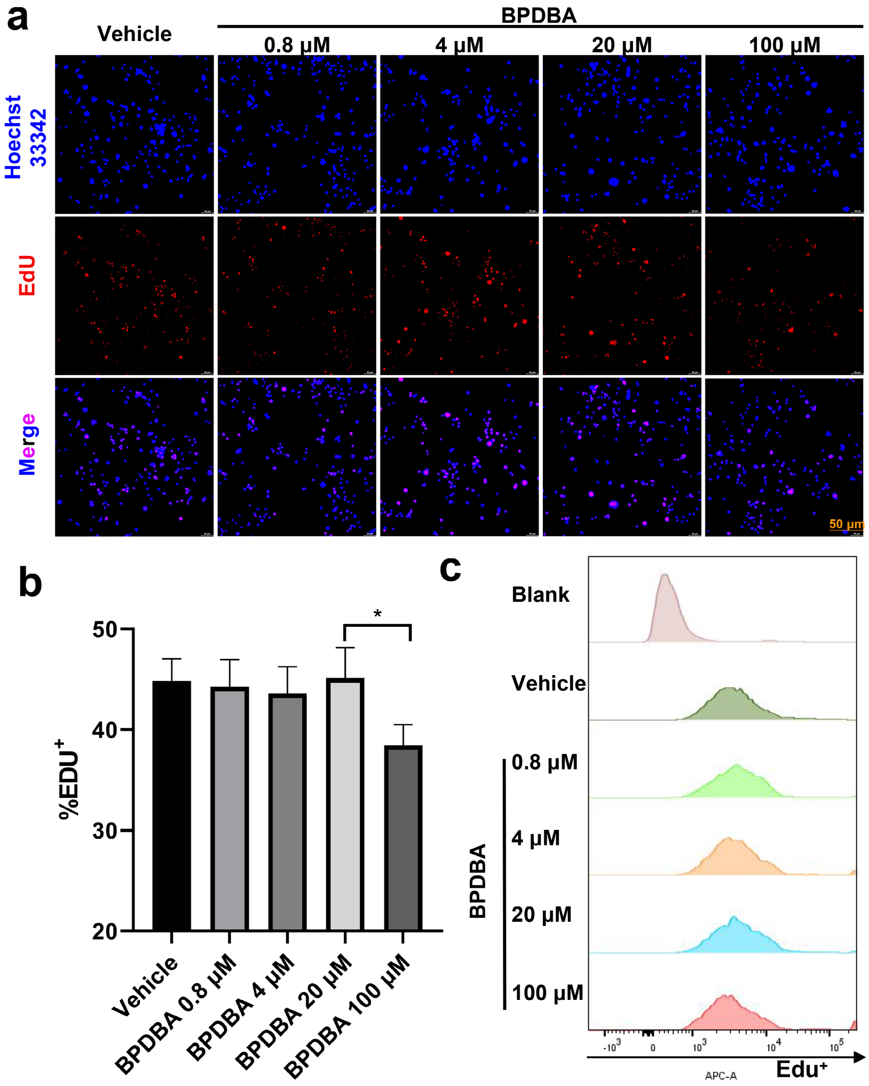

Supplementary Fig6. Testing the effects of the Slc6a12 specific inhibitor BPDBA on the proliferation of THP-1 derived macrophages. (a). Representative images showing the effect of different concentrations of BPDBA on THP-1 cell proliferation using EDU and Hochest fluorescence staining, scale bar=50μm. **(b)**. Statistical chart of the EDU positive rate. (c). Histogram showing the fluorescence intensity of EDU detected by flow cytometry. Data are expressed as the mean ± SD, n=3. * p<0.05.

sFig 7

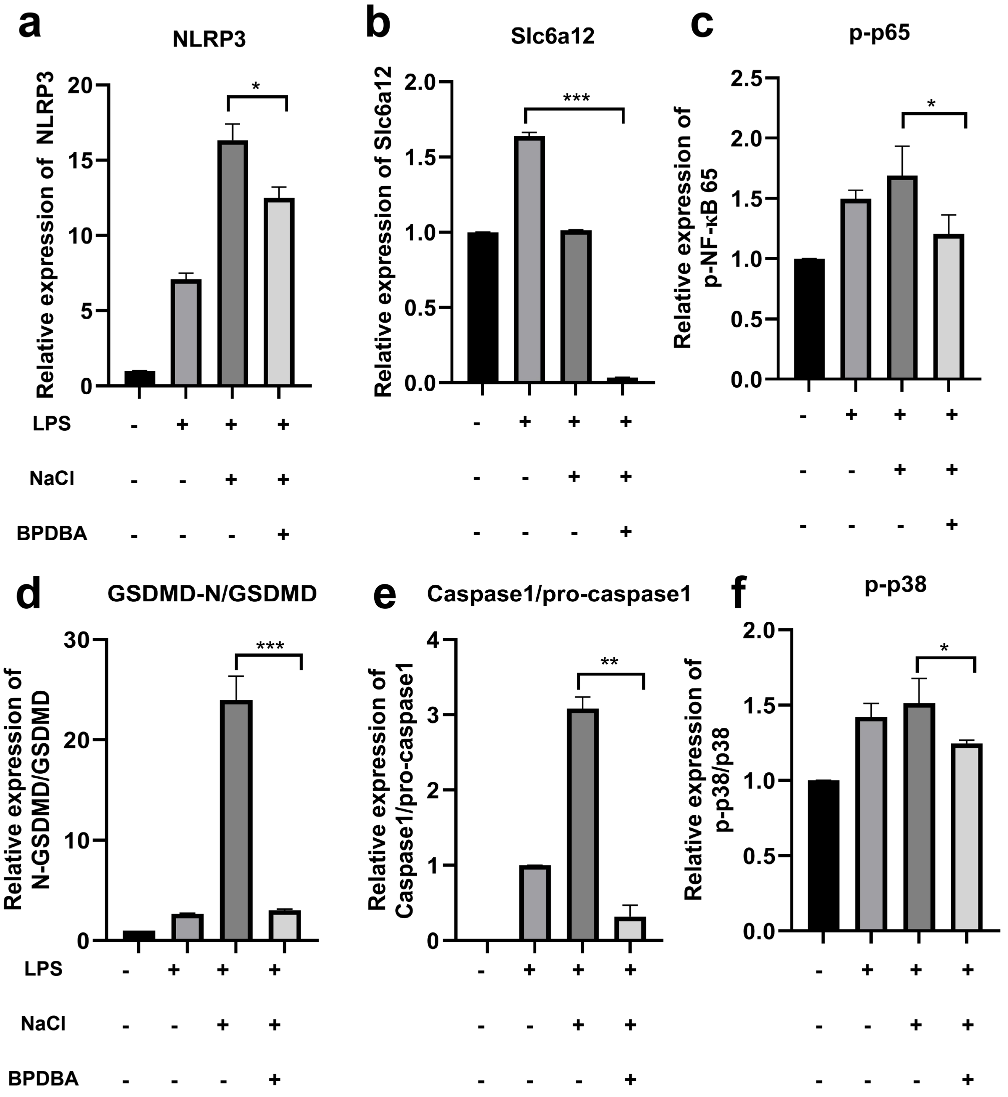

Supplementary Fig7. Quantitative Western blot analysis of the effects of the Slc6a12-specific inhibitor BPDBA on the expression of pyroptosis related proteins in THP-1 derived macrophages. (a-f) Quantitative Western blot analysis of the effects of 20 μM BPDBA on the expression of proteins NLRP3 **(a)**, Slc6a12 **(b)**, p-P65 **(c)** ,GSDMD-N **(d),** caspase1 **(e)** and p38 **(f)** in THP-1 macrophages. Data are expressed as the mean ± SD, n=3. * p<0.05.** p<0.01.*** p<0.001.

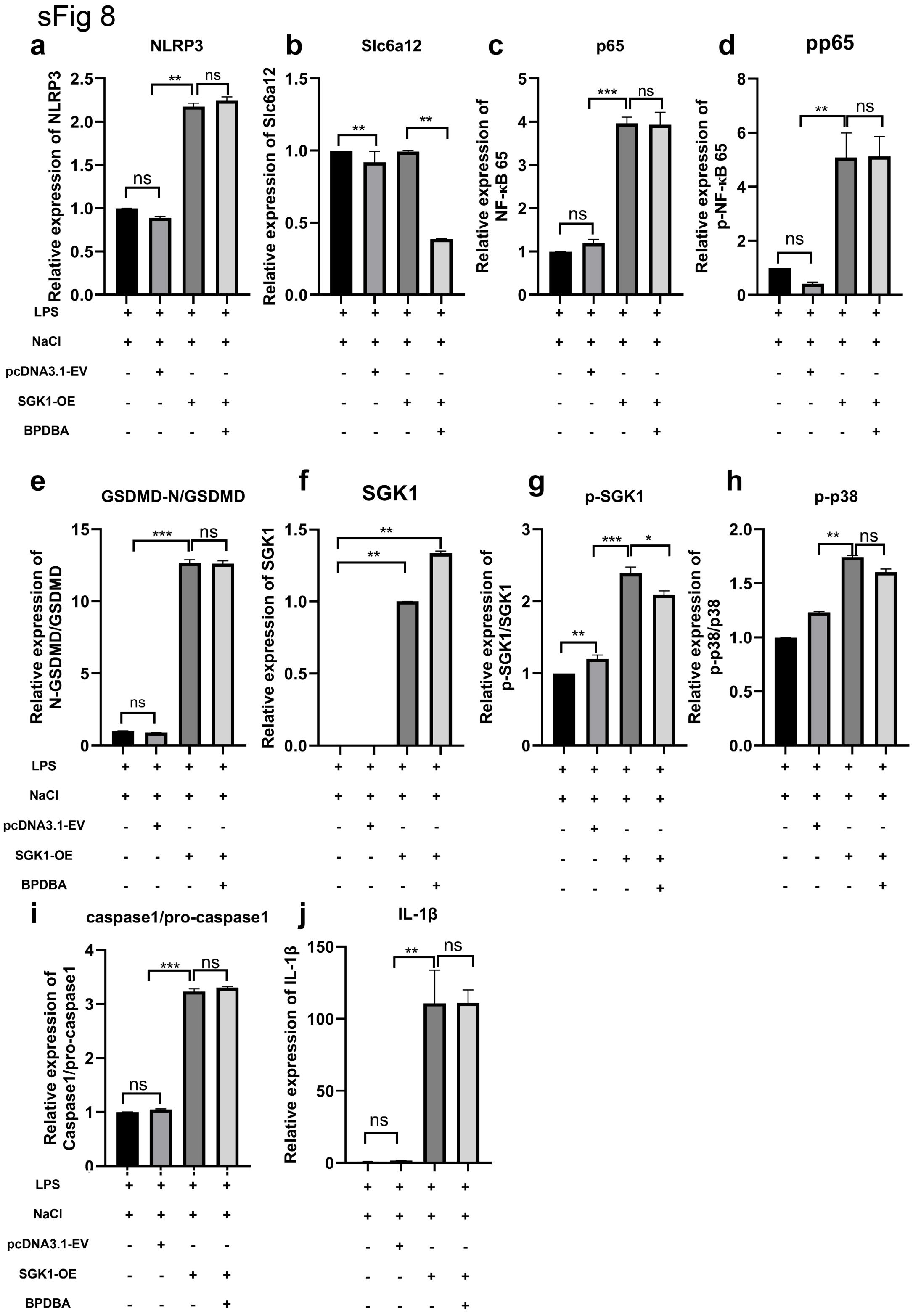

Supplementary Fig8. Quantitative Western blot analysis of the impact of restoring SGK1 expression in SGK1 knockout THP-1 macrophages under NaCl and LPS co-treatment conditions.

(a-j) Satistical and quantitative analysis of the gray scale of Western blot bands of proteins NLRP3 **(a)**, Slc6a12**(b)**, p65**(c)**, p-65**(d)**, GSDMD/GSDMD-N**(e)**, SGK1**(f)**, p-SGK1**(g)**, p-p38/p38**(h)**, caspase1/pro-caspase1**(i)** and IL-1β **(j)** by software Image J. Data are expressed as the mean ± SD, n=3. * p<0.05.** p<0.01.*** p<0.001. ns represent no statistical significance.
